# METTL14 suppresses the expression of YAP1 and the stemness of triple-negative breast cancer

**DOI:** 10.1186/s13046-024-03225-2

**Published:** 2024-11-20

**Authors:** Xupeng Bai, Jiarui Liu, Shujie Zhou, Lingzhi Wu, Xiaojie Feng, Pumin Zhang

**Affiliations:** 1 Zhejiang Provincial Key Laboratory of Pancreatic Disease, The First Affiliated Hospital of Zhejiang University School of Medicine, No.79 Qingchun Road, Hangzhou, 310003 Zhejiang China; 2 Institute of Translational Medicine, Zhejiang University School of Medicine, Hangzhou, 310058 Zhejiang China; 3 Cancer Center, Zhejiang University School of Medicine, Hangzhou, 310018 Zhejiang China; 4 Department of Gynecologic Oncology, Affiliated Cancer Hospital of Zhengzhou University, Zhengzhou, 450000 Henan China

**Keywords:** TNBC, Stemness, METTL14, m^6^A, YAP1, LSD1

## Abstract

**Background:**

Triple-negative breast cancer (TNBC) has pronounced stemness that is associated with relapse. N^6^-methyladenosine (m^6^A) plays a crucial role in shaping cellular behavior by modulating transcript expression. However, the role of m^6^A in TNBC stemness, as well as the mechanisms governing its abundance, has yet to be elucidated.

**Methods:**

We analyzed proteomic and transcriptomic data derived from breast cancer cohorts, with an emphasis on m^6^A regulators. To unravel the role of m^6^A in TNBC, we employed RNA sequencing, methylated RNA immunoprecipitation sequencing, RNA immunoprecipitation, chromatin immunoprecipitation, and luciferase reporter assays with mesenchymal stem-like (MSL) TNBC models. The clinical relevance was validated using human tissue microarrays and publicly accessible databases.

**Results:**

Our findings indicate that the global level of m^6^A modification in MSL TNBC is downregulated primarily due to the loss of *methyltransferase-like 14* (*METTL14*)*.* The diminished m^6^A modification is crucial for the maintenance of TNBC stemness, as it increases the expression of yes-associated protein 1 (YAP1) by blocking YTH domain-containing family protein 2 (YTHDF2)-mediated transcript decay, thereby promoting the activation of Hippo-independent YAP1 signaling. YAP1 is essential for sustaining the stemness regulated by METTL14. Furthermore, we demonstrated that the loss of *METTL14* expression results from lysine-specific demethylase 1 (LSD1)-mediated removal of histone H3 lysine 4 methylation at the promoter region, which is critical for LSD1-driven stemness in TNBC.

**Conclusion:**

These findings present an epi-transcriptional mechanism that maintains Hippo-independent YAP1 signaling and plays a role in preserving the undifferentiated state of TNBC, which indicates the potential for targeting the LSD1-METTL14 axis to address TNBC stemness.

**Supplementary Information:**

The online version contains supplementary material available at 10.1186/s13046-024-03225-2.

## Background

Triple-negative breast cancer (TNBC) is defined as the absence of estrogen receptor (ER), progesterone receptor (PR), and epidermal growth factor receptor 2 (HER2). While defects in homologous recombination render TNBC more responsive to chemotherapy [1, 2], the risk of early relapse remains higher than that of other subtypes [3], and conventional modalities cannot address the malignancy [4]. Studies based on *BRCA1*-mutant breast epithelial lineages have suggested that TNBC may develop from abnormal differentiation and mesenchymal transition of luminal progenitor cells [2, 5]. Consistently, TNBC is histologically poorly differentiated and displays enhanced stemness compared to other breast cancer subtypes, as evidenced by a higher proportion of CD44^+^CD24^−^ and ALDH^+^ breast cancer stem cells (BCSCs) [6–8]. These cells remain dormant but are equipped with robust self-renewal capabilities and heightened resistance to therapy-induced chromatin damage, ultimately leading to treatment failure [9, 10]. The mesenchymal stem -like (MSL) TNBC subtype, whose transcriptome highly resembles BCSC features, has the worst treatment prognosis [11]. Thus, understanding the regulation of stemness in TNBC is critical for overcoming treatment difficulties, particularly given its reliance on chemo-based regimens.

The Hippo signaling pathway exerts a significant suppressive effect on stem cell differentiation and oncogenesis by regulating YAP1 [12, 13]. Normally, the MST/LATS kinase in the Hippo pathway inactivates YAP1 by promoting its phosphorylation and ubiquitination in response to tissue homeostasis and matrix stiffness signals [14], thereby maintaining the normal size and differentiation status of tissues [15]. However, in TNBC, loss of the Hippo pathway results in hyperactivation of YAP1 signaling, which is essential for maintaining basal-like and stem-like properties [16]. This is in stark contrast to ER^+^ breast cancers, in which YAP1 impairs ER-dependent growth [17, 18], underscoring the regulatory specificity of YAP1 in poorly differentiated breast cancers. Previous studies have investigated the upstream cues that trigger YAP1 hyperactivation [19–22]; however, these efforts focused on the mechanisms that silence the Hippo pathway, with the epigenetic control of YAP1 in cancer being largely unknown.

N^6^-methyladenosine (m^6^A) is the predominant form of modification in mammalian mRNA. It is a dynamic and reversible process, governed by the 'writer' complex and demethylases [23, 24]. Various readers recognize the m^6^A mark and exert control over mRNA transcription [25], splicing [26], subcellular distribution [27], stability [28], and translation [29], thereby affecting gene expression and biological processes including carcinogenesis [30]. Methyltransferase-like 14 (METTL14) assumes the role of a navigator in the heterodimer complex with METTL3, responsible for identifying structure-specific m^6^A motifs and guiding METTL3 to accomplish the N^6^-methylation of adenosine [31]. Several clinical studies have highlighted the correlation between low intratumoral *METTL14* expression and unfavorable prognosis in patients with TNBC [32, 33]; however, the exact role and regulatory mechanism have not been addressed.

Here, we unveil the role of m^6^A in steering the stemness of TNBC. Our findings show that the global level of m^6^A is markedly downregulated in TNBC primarily due to the loss of *METTL14*. The enforced *METTL14* expression suppresses TNBC stemness both in vitro and in vivo. Mechanistically, the decreased m^6^A impedes YTHDF2-mediated degradation of *YAP1* transcript, leading to increased expression and transactivation of YAP1, independent of the Hippo pathway. Additionally, we identified histone demethylation-mediated transcriptional repression as a key mechanism underlying the loss of *METTL14* in TNBC.

## Materials and methods

### Cell lines

Breast cancer cell lines used in this study were obtained from the ATCC. MDA-MB-231 and Hs578T cells were grown in Dulbecco's modified Eagle's high-glucose medium (Gibco, China) supplemented with 10% fetal bovine serum (FBS, Vazyme, China). MDA-MB-436, SUM159, MCF7, T47D, BT474, and ZR-75–1 cells were cultured in RPMI-1640 medium (Gibco) supplemented with 10% FBS. All cell lines were maintained in a humidified environment at 37 °C with 5% CO_2_.

### Mammosphere formation assay

Single cells were seeded in ultra-low attachment 96-well or 24-well plates (Corning, USA) at a density of either 1 × 10^3^ or 8 × 10^3^ cells per well using MammoCult™ Human Medium (STEMCELL Technologies, Canada) supplemented with 4 µg/mL heparin (STEMCELL Technologies), and 0.48 µg/mL hydrocortisone (STEMCELL Technologies). Cells were cultured for 4–5 days to form primary spheres. Primary spheres (> 70 μm) were collected and dissociated using 0.25% trypsin–EDTA (Biosharp) for secondary sphere formation. The spheres were counted and photographed using a light field microscope (Olympus, Japan) with a phase-contrast module. Mammosphere formation efficiency (MFE) was calculated as the number of spheres per well divided by the number of cells seeded per well multiplied by 100%.

### Short interference RNA (siRNA) transfection

The siRNAs used in this study were obtained from GenePharma (Suzhou, China). Cells were transfected with siRNAs using Lipofectamine™ RNAiMAX Transfection Reagent (Invitrogen, USA) and Opti-MEM (Gibco) for a period of 48–72 h. Knockdown efficiency was verified by immunoblotting. Detailed information regarding the target sequences of the siRNAs is provided in Suppl. Table 1.

### Lentiviral transduction

Stable depletion of *METTL14* was accomplished using lentiviruses carrying DNA oligos encoding specific short hairpin RNAs (shRNAs). These lentiviruses were generated through the co-transfection of HEK293T cells with *pMD2.G*, *psPAX2*, and *pLKO.1* (sh*METTL14*, sh*YAP1*, or sh*LSD1*) plasmids, and concentrated using a Universal Virus Concentration Kit (Beyotime, China). The *pLKO.1-shcontrol* (shNC) served as the negative control. Detailed information regarding the target sequences of the shRNAs is provided in Suppl. Table 1. Stable expression of *METTL14* was achieved using lentiviruses harboring either the wild-type or mutant *METTL14* coding sequence (CDS). These lentiviruses were produced through the co-transfection of HEK293T cells with *pMD2.G*, *psPAX2*, *pCDH-M14-WT* (or *pCDH-M14-R298P*) plasmids and concentrated using a Universal Virus Concentration Kit. The *pCDH* vector was used as the empty control. Cells were transduced with lentivirus in a culture medium supplemented with 8 µg/mL polybrene (Beyotime). After 48 h, the transduced cells were cultured in virus-free medium containing 4 μg/mL puromycin (Beyotime) for 48-h selection and then maintained in medium containing 2 μg/mL puromycin. Knockdown efficiency was verified by immunoblotting.

### Plasmid transfection

The CDS of the gene responsible for expressing a specific protein was synthesized by Sangon Biotech (Shanghai, China) and inserted into the multiple cloning site (MCS) of the *pCDH* vector. Ligated plasmids were obtained by agarose gel extraction and subjected to antibiotic selection. The endotoxin-free plasmid was introduced into cells using Lipofectamine 2000 Transfection Reagent (Invitrogen) and Opti-MEM (Gibco) and incubated for a period of 48–72 h. Protein expression was confirmed by immunoblotting.

### Immunoblotting

Protein lysates were extracted using RIPA Lysis and Extraction Buffer (Thermo Fisher Scientific) supplemented with 1 × Halt Protease and Phosphatase Inhibitor Cocktail (Thermo Fisher Scientific). Nuclear and cytoplasmic proteins were extracted using a Nuclear and Cytoplasmic Protein Extraction Kit (Beyotime). Protein samples were quantified using the Pierce BCA Protein Assay Kit (Thermo Scientific) and denatured in 1 × SDS-PAGE Sample Loading Buffer (Beyotime). For immunoblot analysis, equal amounts of protein (10–20 μg) were separated on a 10% SDS-PAGE gel, transferred to a 0.45 μm Immobilon polyvinylidene fluoride membrane (Millipore, USA), and then blocked with 5% skim milk. After overnight incubation with the primary antibody at 4 °C, the membrane was incubated with horseradish peroxidase (HRP) -conjugated secondary antibody for 1 h at room temperature (RT). The protein lanes were detected using the SuperSignal West Pico PLUS Chemiluminescent Substrate (Thermo Fisher Scientific) and captured using an ImageQuant 800 Western blot CCD imager (Cytiva, Amersham, UK). GAPDH was used as the internal reference for total or cytoplasmic protein, while Lamin B was used for nuclear protein. Information regarding the antibodies used is provided in Suppl. Table 2.

### Immunohistochemical (IHC)

The human TNBC tissue microarray was obtained from Weiao Biotechnology (Shanghai, China). Human tissue microarray for luminal breast cancer was obtained from the tissue bank of the Department of Gynecologic Oncology, Affiliated Cancer Hospital of Zhengzhou University. The slides were deparaffinized with xylene and dehydrated with ethanol. After antigen retrieval with 0.01 M citrate buffer, the sections were blocked with goat serum and incubated with primary antibody at 4 °C overnight. Next, the sections were incubated with HRP-conjugated anti-rabbit secondary antibody for 1 h at RT and stained with the DAB HRP Color Development Kit (Beyotime). The IHC score was determined as the product of the percentage of positive cells and staining intensity (weak = 1; moderate = 2; strong = 3). Information regarding the antibodies used is provided in Suppl. Table 2.

### Quantitative real-time PCR (qRT-PCR)

Total RNA was extracted using the EZ-press RNA Purification Kit (EZBioscience, USA). RNA quality was measured using a NanoDrop One Microvolume UV–Vis Spectrophotometer (Thermo Fisher Scientific). Reverse transcription was performed with 1 μg total RNA using the Script cDNA Synthesis Kit (Bio-Rad, USA), and qRT-PCR was performed with 100 ng cDNA using the SYBR Green Supermix (Bio-Rad) on a CFX96 Real-Time PCR Detection System (Bio-Rad). GAPDH was used as the internal reference. Relative gene expression was calculated using the 2^−ΔΔCT^ method. Primers used for qRT-PCR are listed in Suppl. Table 3.

### Dot blotting

The relative level of mRNA m^6^A modification was measured by dot blotting. Total mRNA was prepared using the Mag-MK mRNA Purification Kit (Sangon Biotech) and boiled for 3 min at 95 °C. The samples (200, 400, or 800 ng) were then loaded onto nitrocellulose membranes, cross-linked with UV light, and blocked with 5% skim milk. After overnight incubation with a primary antibody against N^6^-methyladenosine (#56593, CST, USA) at 4 °C, the membrane was incubated with HRP-conjugated anti-rabbit secondary antibody (#7074, CST) for 1 h at RT and stained with 1 × methylene blue solution (Sangon Biotech) for 2 h at RT. The membrane was detected using the SuperSignal West Pico PLUS Chemiluminescent Substrate (Thermo Fisher Scientific) and captured using an ImageQuant 800 Western blot CCD imager.

### Flow cytometry analysis

Cell suspensions were filtered through a 37-μm filter and resuspended in flow cytometry staining buffer (Proteintech, China). Cells were stained with BODIPY-aminoacetaldehyde (STEMCELL Technologies) or fluorescence-conjugated antibodies against BCSC surface markers and analyzed using a CytoFLEX LX Flow Cytometer (Beckman, USA). Information regarding the antibodies used is provided in Suppl. Table 2. Data were processed using FlowJo v10.8.1 software (BD Biosciences, USA).

### Chemo-/radio-sensitivity assay

The chemo-/radiosensitivity of cells was assessed by colony formation assay. Cells were seeded in 6-well plates and treated with different concentrations of doxorubicin or paclitaxel for 48 h, or with a single dose of irradiation. Then these cells were cultured for 7–9 days and stained with a 0.5% crystal violet solution. The colonies (> 50 cells) were counted and photographed.

### RNA immunoprecipitation (RIP)

Cells were collected in Pierce IP lysis buffer (Thermo Fisher Scientific) containing 1 × Halt Protease and Phosphatase Inhibitor Cocktail and 0.1 U/μL RNase inhibitor (Beyotime). Cell lysates were incubated with 4 μg anti-YTHDF2 (24,744–1-AP, Proteintech) or IgG control antibody (30,000–0-AP, Proteintech) at 4 °C overnight in RIP buffer (150 mM KCl, 25 mM Tris, 5 mM EDTA, 0.5 mM DTT, 0.2% IGEPAL CA-630, 1 × Halt Protease and Phosphatase Inhibitor Cocktail and 0.1U/μL RNase inhibitor) with rotation. The samples were then mixed with 50 μL Pierce Protein A/G Magnetic Beads (Thermo Fisher Scientific) at 4 °C for 2 h to capture the antibody-protein-RNA complex. The beads were resuspended in 150 μL RIP buffer containing 0.1% SDS and incubated with 30 μg proteinase K (Beyotime) at 55 °C for 30 min to remove proteins. The supernatant was subjected to RNA extraction and qRT-PCR. Primers used for RIP-qPCR are listed in Suppl. Table 3.

### RNA stability assay

Cells were treated with 5 μg/mL Actinomycin D (Act.D, GlpBio, USA) for 0, 2, 4, 6, and 8 h, and total RNA was extracted using the EZ-press RNA Purification Kit and used for qRT-PCR. The percentage of remaining mRNA was determined as the average Cq at each time point relative to the average Cq at 0 h. The decay rate of the mRNA is shown as an exponential fit.

### Protein stability assay

Cells were treated with 100 μg/mL cycloheximide (CHX; CST, USA) for 0, 1, 3, 6, and 9 h, and total protein was extracted and subjected to immunoblotting.

### RNA sequencing (RNA-seq) and methylated RIP-sequencing (MeRIP-seq)

For RNA-seq, poly(A) mRNA was extracted using the Dynabeads® mRNA DIRECT™ Kit (Thermo Fisher Scientific), and library preparation was performed using the NEBNext® Ultra™ RNA Library Prep Kit (New England Biolabs, USA). Each group was sequenced in duplicate by Illumina NovaSeq 6000 with pair-end 75 cycles. Raw reads were obtained from the sequencing machine and cleaned using fastp v0.18.0 to remove adapters and low-quality bases. Clean reads were aligned to the human reference genome GRCh37/hg19 using HISAT v2.2.4. Differentially expressed genes (DEGs) were analyzed using DESeq2 v1.40.2 with a threshold (*p* < 0.05 and fold change > 1.5). For MeRIP-seq, poly(A) mRNA was extracted and fragmented using Ambion® RNA Fragmentation Reagents (Thermo Fisher Scientific). m^6^A immunoprecipitation was performed using fragment RNA and m^6^A monoclonal antibody (#68,055–1-Ig, Proteintech) according to the RIP procedure. RNA from the input and m^6^A IP samples was purified using the BeyoMag™ mRNA Purification Kit (Beyotime) and used for library preparation using the NEBNext® Ultra™ RNA Library Prep Kit. The input and m^6^A IP libraries were sequenced by Illumina HiSeq 4000 with 150 bp paired-end reads. Sequencing data were deposited in the NCBI Gene Expression Omnibus under accession code GSE245282. Raw reads were cleaned and aligned to the human reference genome GRCh37/hg19. Peak calling and annotation were performed using MACS2 v2.1.2 (*q* < 0.05) and exomePeak2 v1.12.0 (*P* < 0.00001). The base frequency matrix and motif search were accomplished with Homer v3.0. DiffBind v2.8 software was used to merge peaks among groups, and exomePeak2 was used to obtain the levels of merged peaks in each sample. Differential peaks were identified with FDR < 0.05 and fold change > 2. Sequencing data were deposited in the NCBI Gene Expression Omnibus (GEO) database under accession number GSE245282. Genes of interest were visualized using Integrative Genomics Viewer v2.16.2. Pathway enrichment analysis was performed with the KEGG database. Gene set enrichment analysis was performed with GSEA v4.3.2 and molecular signature databases (https://www.gsea-msigdb.org).

### Dual-luciferase reporter assay

Transcriptional activity of *YAP1* was measured by dual-luciferase reporter assay. To this end, the promoter region (-800/+100) of *YAP1* was cloned upstream of the modified *Firefly luciferase* (F-luc) gene into the *pGL3-basic* vector (Promega, USA) and co-transfected with *pRL-TK* expressing Renilla luciferase (R-luc). Relative light unit (RLU) was detected 48 h after transfection using a Dual-Luciferase Reporter Gene Assay Kit (Beyotime) on a microplate reader (BioTek Synergy 2, USA). Transcriptional activity was determined by the RLU ratio of firefly to renilla. The translational efficiency of *YAP1* mRNA was measured using the *pmirGLO* vector (Promega) by inserting the exon 10 and 3’UTR of the *YAP1* gene downstream of the *F-luc* gene. The ratio of F-luc to R-luc (reporter protein expression) was determined 24 h after transfection using a Dual-Luciferase Reporter Gene Assay Kit on a multifunctional microplate reader, while the mRNA expression ratio of F-luc to R-luc (reporter mRNA expression) was determined by qRT-PCR. The primers used for reporter mRNA analysis are listed in Suppl. Table 3. Translation efficiency is calculated by the ratio of reporter protein level to mRNA level. The m^6^A modification sites were investigated using the *pmirGLO* vector by inserting the exon 10 region of *YAP1* containing the wild-type or mutant m^6^A motif (A was replaced by T) downstream of the *F-luc* gene. The effect of the point mutation on reporter protein expression was determined by luminescence detection 24 h after transfection.

### Chromatin immunoprecipitation (CHIP)

The CHIP assay was performed using the BeyoChIP Enzymatic ChIP Assay Kit (Beyotime). Cellular genomes and proteins were cross-linked with 1% formaldehyde and collected in 1 × PBS supplemented with 1 × Halt Protease and Phosphatase Inhibitor Cocktail. The genome was digested and fragmented in CHIP buffer containing micrococcal nuclease, and the fragmentation effect was validated by agarose gel electrophoresis. Fragmented samples were mixed with 4 µg anti-H3K4me2 (91,322, Proteintech) or IgG control antibody (30,000–0-AP, Proteintech) overnight at 4 °C with slow rotation, followed by incubation with 30 µL Protein A/G Magnetic Beads/Salmon Sperm DNA with gentle rotation for 3 h at 4 °C. The magnetic beads were eluted with an Elution Buffer to collect the supernatant. The supernatant was heated at 65 °C for 2 h to remove cross-linking and incubated with 0.15 mg/mL proteinase K at 45 °C for 60 min to remove the protein for subsequent RNA extraction and qRT-PCR. Primers used for CHIP-qPCR are listed in Suppl. Table 3.

### Animal experiments

Female athymic (nu/nu) BALB/C mice (5-week old, 18–20 g) were purchased from Gempharmatech Co. Ltd. (Jiangsu, China). The mice were housed in a specific-pathogen-free (SPF) animal facility at the First Affiliated Hospital of Zhejiang University School of Medicine and allowed free access to SPF-grade food and water. All animal procedures were approved by the Animal Care and Use Committee of the First Affiliated Hospital of Zhejiang University School of Medicine. After one-week adaptation, the mice were randomly divided into the indicated groups with five mice per group. Gene-modified cells in the logarithmic growth phase were collected and subcutaneously injected into the right flank of mice at the indicated density in 0.1 mL 1 × PBS to perform the limiting dilution assay (LDA). Tumor size was measured using a caliper every four days, and tumor volume was calculated according to the following formula (length × width^2^ × 0.5). Tumor incidence was recorded every 4 days, and the frequency of CSCs/CICs in tumors was analyzed using the L-Calc™ software (STEMCELL Technologies).

### Database analysis

Intratumoral protein expression among the different breast cancer subtypes was analyzed using The Cancer Genome Atlas (TCGA) Breast Cancer Proteome (PDC000173) and Clinical Proteomic Tumor Analysis Consortium (CPTAC) Prospective Breast BI Proteome (PDC000120) from the NCI Proteomic Data Commons. Intratumoral gene expression among different breast cancer subtypes was analyzed using TCGA-BRCA dataset from the NCI GDC Data Portal. The correlation between *METTL14* expression and the tumor stemness index was evaluated by Pearson’s correlation analysis using the uniformly normalized TCGA pan-cancer datasets from the UCSC Xena and the tumor stemness indices calculated by DNA methylation signatures for each sample from the NCI Genomic Data Commons [34].

### Statistical analysis

Data are presented as the mean ± SD derived from three biological replicates. Statistical analyses were performed using the Prism 9 software (GraphPad, USA). The differences between two groups were assessed using an unpaired two-tailed Student’s t-test. For multiple comparisons, one-way or two-way analysis of variance (ANOVA) accompanied by Šídák post hoc testing was employed. Statistical significance was set at* P* < 0.05.

### Role of funders

The funder provided financial support for the study reagents, materials, and salaries of assistant personnel; however, they did not participate in the implementation of the study or dissemination of findings.

## Results

### Loss of *METTL14* correlates with TNBC stemness

**Fig. 1:**
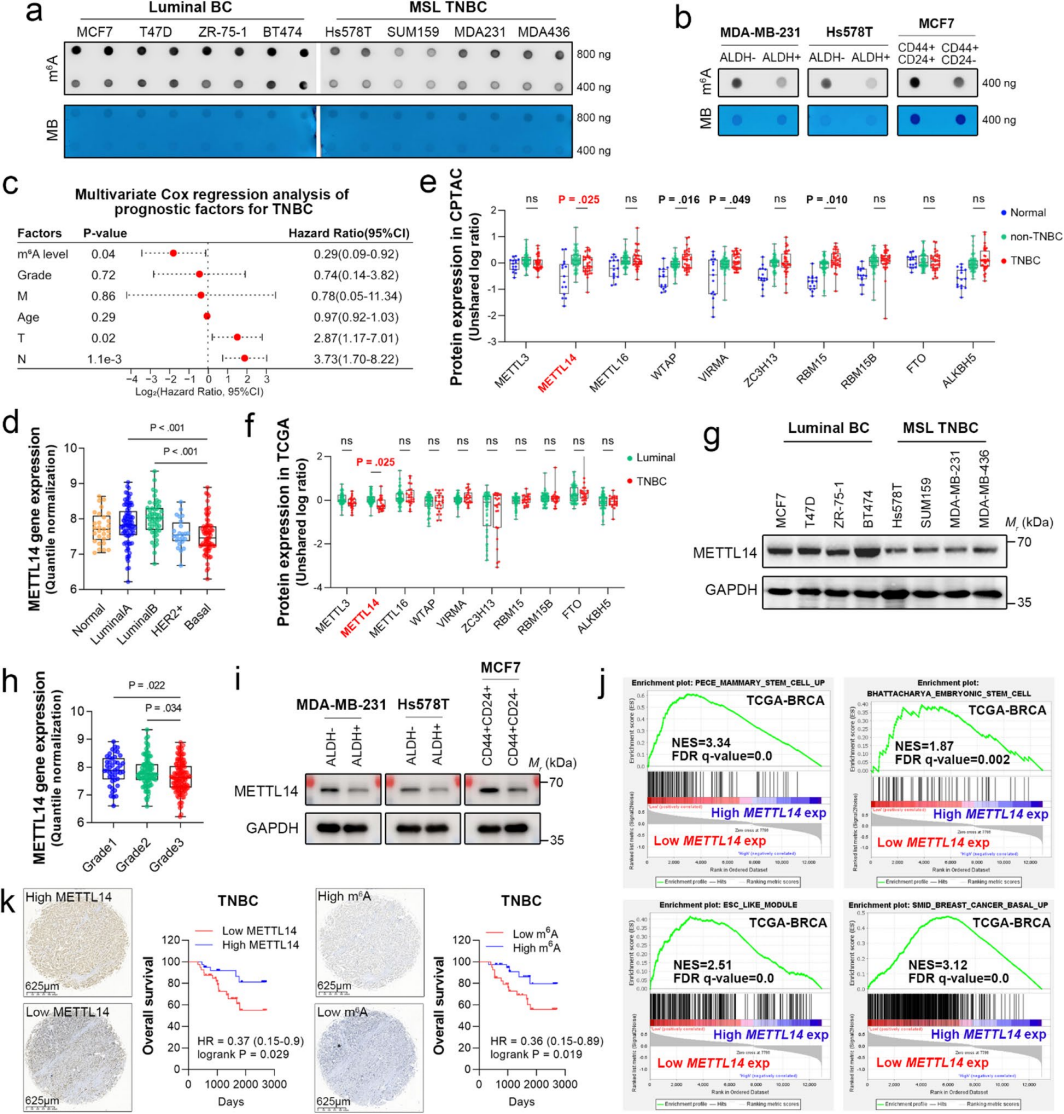
Loss of *METTL14* correlates with TNBC stemness. **a**, **b** Global m^6^A levels of mRNA in representative cell lines of human luminal breast cancer (BC), mesenchymal stem cell-like (MSL) TNBC, ALDH^+^ or CD44^+^CD24^−^ breast cancer stem cells (BCSCs), and non-BCSCs were determined using dot blotting. Methylene blue (MB) staining was used as the loading control. **c** Multivariable Cox regression analysis was conducted to determine the hazard ratio for each prognostic factor of TNBC (*n* = 80 from the human tissue microarray). **d** Expression of *METTL14* in normal adjacent (*n* = 29), luminal A (*n* = 89), luminal B (*n* = 49), HER2^+^ (*n* = 24), and basal (*n* = 75) tumor tissues was obtained from the GSE21653 cohort. Data are shown as the mean ± SD of the normalized microarray expression. **e, f** Protein expression of METTL3, METTL14, METTL6, WTAP, VIRMA, ZC3H13, RBM15, RBM15B, FTO, and ALKBH5 in normal adjacent (*n* = 15), non-TNBC (*n* = 84), TNBC (*n* = 35), luminal breast cancer (*n* = 70), and TNBC (*n* = 23) tissues was obtained from the Clinical Proteomic Tumor Analysis Consortium (CPTAC) and the Cancer Genome Atlas (TCGA). Data are shown as the mean ± SD of normalized protein expression. **g** Protein expression of METTL14 in representative cell lines of luminal BC and MSL TNBC was determined using immunoblotting. GAPDH was used as the loading control. **h** Expression of *METTL14* in Scarff-Bloom-Richardson grade1 (*n* = 45), grade2 (*n* = 89), and grade3 (*n* = 125) tissues was obtained from the GSE21653 cohort. Data are shown as the mean ± SD of the normalized microarray expression. **i** Protein expression of METTL14 in BCSCs and non-BCSCs was determined using immunoblotting. GAPDH was used as the loading control. **j** Enrichment plots show the gene set enrichment analysis of the whole transcriptome between *METTL14* low expression (bottom 25%, *n* = 272) and high expression (top 25%, *n* = 272) groups in TCGA-BRCA cohort using various stemness-related gene signatures. **k** Representative images of immunohistochemistry (IHC) staining for METTL14 and m^6^A in human TNBC tissues. Scale bars: 625 μm. Survival analysis of TNBC patients (*n* = 80, from the tissue microarray) stratified by METTL14 or m^6^A IHC score (median) was conducted using the log-rank test

We found that MSL TNBC cell lines displayed lower global m^6^A levels than luminally differentiated ER^+^ breast cancer cell lines (MCF7 and T47D) and ER^+^HER2^+^ breast cancer cell lines (ZR-75–1 and BT474) (Fig. 1a). We reasoned that the diminished m^6^A might be associated with poor differentiation status of breast cancer. CD44^+^CD24^−^ or ALDH^+^ breast cancer stem cells (BCSCs) isolated from multiple breast cancer cell lines confirmed these findings (Fig. 1b and Suppl. Figure 1a). Multivariate Cox regression analysis of prognostic factors for TNBC identified the intratumoral m^6^A level as an independent factor for overall survival (OS) (HR = 0.29, 95% CI 0.09–0.92, *P* = 0.04) **(**Fig. 1c**)**. To determine the underlying mechanism, we analyzed the gene expression of known m^6^A regulators in breast cancer tissues from The Cancer Genome Atlas (TCGA) and found that *METTL14* expression was much lower in TNBC than in normal adjacent and non-TNBC tissues, as compared to other regulators (Suppl. Figure 1b). Similar results were also obtained from GSE21653 [35] **(**Fig. 1d**)**. We next consulted proteomics data from the Clinical Proteomic Tumor Analysis Consortium (CPTAC) and TCGA-BRCA and found that METTL14 was significantly downregulated in TNBC tissues compared with non-TNBC tissues (Fig. 1e and f). Consistently, the protein expression of METTL14 was diminished in multiple MSL TNBC cell lines compared with that in luminal breast cancer cells (Fig. 1g). Low expression of *METTL14* correlated with a higher Scarff-Bloom-Richardson grade (Fig. 1h). Pan-cancer analysis of the tumor stemness index showed that *METTL14* expression exhibited the most significant negative correlation with the stemness of breast cancer (r = -0.14, *P* < 0.0001) [34] (Suppl. Figure 1c). Immunoblotting of METTL14 in CD44^+^CD24^−^ or ALDH^+^ BCSCs confirmed this correlation (Fig. 1i). Furthermore, gene set enrichment analysis using the transcriptome from TCGA-BRCA cohort presented a notable enrichment of stem/basal gene signatures in the *METTL14* low-expression group [36–39] (Fig. 1j). Survival analysis according to IHC scores of tumor tissues showed that patients with low levels of METTL14 or m^6^A had a shorter OS duration (Fig. 1k), and this prognostic correlation was also demonstrated by KMplot analysis (Suppl. Figure 1d). These results suggest that the loss of *METTL14* correlates with poor differentiation status of breast cancer and may play a role in maintaining the stemness of TNBC.

### METTL14-mediated m^6^A modification suppresses the stem-like trait of TNBC

**Fig. 2:**
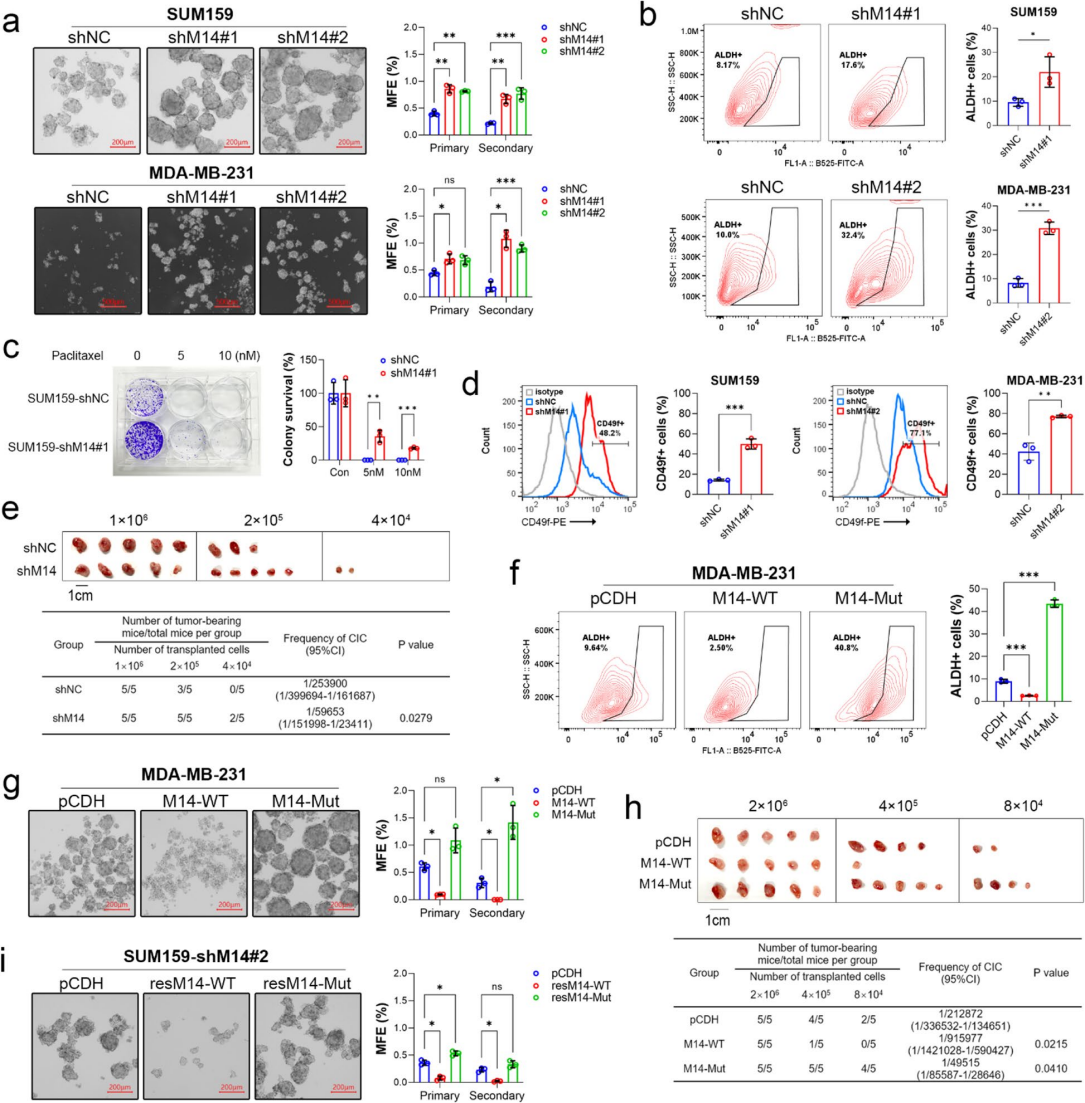
METTL14-mediated m^6^A modification suppresses the stem-like trait of TNBC. **a** Representative images of the secondary mammospheres of TNBC cells with or without *METTL14* depletion. Scale bar: 200 μm (upper panel) and 500 μm (lower panel). **b** Flow cytometric analysis of ALDH^+^ cell population in SUM159 and MDA-MB-231 cells with or without *METTL14* depletion. **c** Representative images of colonies formed by SUM159 shNC and shM14 cells treated with paclitaxel (PTX) for 48 h. **d** Flow cytometric analysis of CD49f^+^ cell population in SUM159 and MDA-MB-231 cells with or without *METTL14* knockdown. **e** The image shows the number of tumor-bearing mice in the indicated group (*n* = 5 per group) after inoculating BALB/C mice with shM14 or shNC MDA-MB-231 cells. The cancer-initiating cell (CIC) frequency was quantified using the L-Calc software. **f** Flow cytometric analysis of ALDH^+^ cell population in MDA-MB-231 cells overexpressing wild-type (WT) or mutated *METTL14*. **g** Representative images of the secondary mammospheres of MDA-MB-231 cells overexpressing WT or mutated *METTL14*. **h** The image shows the number of tumor-bearing mice in the indicated group (*n* = 5 per group) after inoculation of BALB/C mice with MDA-MB-231 cells overexpressing WT or mutated *METTL14*. Quantification of CIC frequency was performed using L-Calc software. **i** Representative images of the secondary mammospheres of *METTL14*-depleted SUM159 cells expressing shRNA-resistant WT or mutated *METTL14*. Data are shown as the mean ± SD for a representative of three independent experiments performed in triplicate. *P < 0.05; **P < 0.01; ***P < 0.001; ns, nonsignificant

To determine the role of METTL14, we established *METTL14*-depleted MCF7, MDA-MB-231, and SUM159 cell lines using a lentivirus-mediated hairpin RNA (shRNA) expression system with two different shRNAs targeting *METTL14*. These stable cell lines displayed efficient depletion of *METTL14* (Fig. 4a and b) and global m^6^A compared to cells transduced with the negative control lentivirus (Suppl. Figure 2a). Mammosphere formation under ultralow attachment culture conditions showed that *METTL14* depletion significantly promoted both primary and secondary sphere formation in SUM159 and MDA-MB-231 cells (Fig. 2a). The depletion increased the percentage of ALDH^+^ BCSCs in SUM159 and MDA-MB-231 cells (Fig. 2b). Chemoresistance is an important hallmark of BCSCs. *METTL14* depletion not only enhanced cell resistance to paclitaxel (Fig. 2c) and doxorubicin (Suppl. Figure 2b) but also conferred radioprotective effects on these cells (Suppl. Figure 2c). Furthermore, the proportion of CD49f^+^ basal-like cells, which mark a BCSC population associated with taxane resistance in TNBC [40], was also increased by *METTL14* knockdown (Fig. 2d). We next inoculated immunodeficient mice with decreasing numbers of stable shNC and shM14 cells and evaluated tumor incidence using limiting dilution analysis (LDA) [41]. The results showed that as few as 4 × 10^4^ *METTL14*-depleted TNBC cells were capable of forming tumors in 2/5 immunodeficient mice compared to 0/5 for control cells (Fig. 2e), suggesting that *METTL14* depletion could enrich CSCs in TNBC.

METTL14 is the core component of m^6^A-installing methyltransferase heterodimer. To investigate whether the role of METTL14 in TNBC is associated with its canonical function, we overexpressed wild-type *METTL14* (M14-WT) or a loss-of-function mutant (M14-Mut, R298P) in MDA-MB-231 cells (Suppl. Figure 2d and 4c). The inactive mutant robustly increased the proportion of ALDH^+^ cells, whereas the wild-type *METTL14* reduced this proportion (Fig. 2f). Apparently, the mutant acted in a dominant-negative manner. Similarly, wild-type *METTL14* could effectively suppress primary and secondary sphere formation in MDA-MB-231 cells, whereas the mutant enhanced sphere formation (Fig. 2g). The in vivo LDA also demonstrated that the overexpression of wild-type *METTL14* decreased, while the inactive *METTL14* mutant increased, the tumor incidence (Fig. 2h). In addition, we restored *METTL14* expression in *METTL14*-depleted cells using shRNA-resistant *METTL14* (resM14-WT or resM14-Mut) (Fig. 4b). The re-expression of resM14-WT, but not resM14-Mut, restored global m^6^A level (Suppl. Figure 2a) and reduced the sphere-forming ability of *METTL14*-depleted cells (Fig. 2i), suggesting that shRNAs targeting *METTL14* were on target. These results demonstrate that METTL14 suppresses the stemness of TNBC via its writer function.

### Identification of the m^6^A-regulated transcripts involved in TNBC stemness

**Fig. 3:**
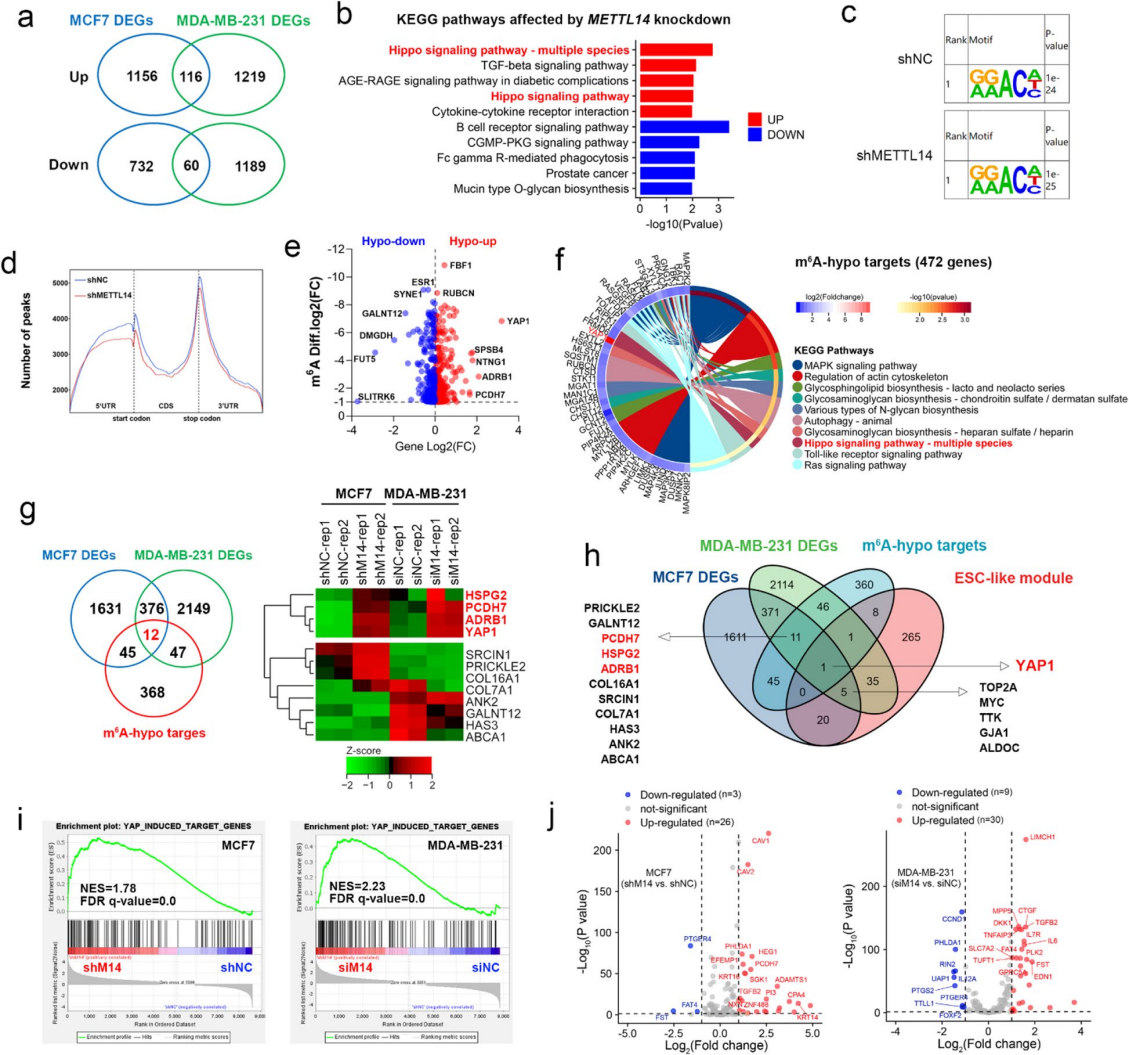
Identification of m^6^A-regulated transcripts involved in TNBC stemness. **a** Venn diagrams show the number of significantly upregulated or downregulated genes in MCF7 and MDA-MB-231 cell lines (*P* < 0.05, fold change ≥ 1.5) upon *METTL14* depletion. Intersection shows the number of shared differentially expressed genes (DEGs). **b** Bar chart shows the top five upregulated or downregulated Kyoto Encyclopedia of Genes and Genomes (KEGG) pathways enriched in 176 DEGs. **c** The predominant consensus m^6^A motif identified by the HOMER software in the MeRIP-seq library of MCF7 cells. **d** Metagene plot shows the distribution of m^6^A peaks in different functional regions of mRNA in MCF7 shNC and shM14 cell lines. **e** Volcano plot shows the statistical distribution of m^6^A-hypo genes (diff *P* < 0. 05, fold change > 2) with decreased mRNA expression (hypo-down) and increased mRNA expression (hypo-up) in MCF7 cells upon *METTL14* depletion. **f** Enrichment circle plot shows the top ten KEGG pathways enriched in m^6^A-hypo genes. **g** Venn diagrams show the overlay analysis of shared DEGs with m^6^A-hypo genes upon *METTL14* depletion. Intersection shows the number of shared m^6^A-hypo DEGs. The heatmap shows the mRNA expression of selected genes across all samples for RNA sequencing. **h** Venn diagrams show the overlay analysis of shared m^6^A-hypo DEGs with the core embryonic stem cell (ESC)-like gene module. **i** Enrichment plots show gene set enrichment analysis of the whole transcriptome in MCF7 and MDA-MB-231 cell lines upon *METTL14* depletion using the YAP1-induced target gene set. **j** Volcano plot shows the expression of YAP1-induced target genes in MCF7 and MDA-MB-231 cells upon *METTL14* depletion

To explore the mechanisms by which METTL14-mediated m^6^A modification regulates breast cancer stemness, we performed methylated RNA immunoprecipitation (MeRIP) sequencing using MCF7 cells, in which *METTL14* is highly expressed. We also retrieved transcriptome sequencing data from MDA-MB-231 cells transfected with short interfering RNAs (siRNAs) targeting *METTL14* or control siRNAs from the GSE81164 dataset [42]. A total of 116 upregulated and 60 downregulated genes (*P* < 0.05, fold change ≥ 1.5) shared by MCF7 and MDA-MB-231 cells were identified after *METTL14* depletion (Fig. 3a and Suppl. Data 1). Pathway analysis of these differentially expressed genes using the Kyoto Encyclopedia of Genes and Genomes (KEGG) database showed that METTL14*-*regulated genes were associated with the Hippo signaling pathway (*P* = 0.002) (Fig. 3b and Suppl. Data 1).

In addition, we identified 41,427 m^6^A peaks in the MeRIP sequencing library of MCF7 cells (Suppl. Figure 3a). These m^6^A peaks were significantly enriched in the most common RRACH consensus motif (R, purine; A, methylable adenosine; C, cytosine; H, non-guanine base) (Fig. 3c) and were mainly distributed around the stop codon site of the mRNAs (Fig. 3d and Suppl. Figure 3b). Genes with one or more authentic m^6^A-hypo peaks (diff *P* < 0.05, fold change > 2) after *METTL14* knockdown were termed as m^6^A-hypo genes. A total of 472 m^6^A-hypo genes were identified in *METTL14*-depleted MCF7 cells (Suppl. Data 2). Most of these genes were associated with upregulated mRNA expression following *METTL14* depletion (Suppl. Figure 3c), and *YAP1* was the most significantly upregulated gene in *METTL14*-depleted MCF7 cells (Fig. 3e). KEGG pathway analysis of these m^6^A-hypo genes revealed that the Hippo signaling pathway was greatly affected by m^6^A alterations mediated by METTL14 (*P* = 0.025) (Fig. 3f and Suppl. Data 2), which is in line with the pathway analysis of shared differentially expressed genes. Overlap analysis of the m^6^A-hypo genes and shared differentially expressed genes showed that there were 12 genes potentially regulated by METTL14-mediated m^6^A modification (Fig. 3g). Thereinto, the expression of heparan sulfate proteoglycan 2 (*HSPG2*), protocadherin 7 (*PCDH7*), adrenoceptor β1 (*ADRB1*), and *YAP1* was consistently upregulated in *METTL14*-depleted MCF7 and MDA-MB-231 cells (Fig. 3g). To determine the dominant gene regulating TNBC stemness, we overlapped these genes with the core embryonic stem cell-like gene module and found that *YAP1* was the principal effector of stemness regulated by METTL14-mediated m^6^A modification [37] (Fig. 3h). Consistently, gene set enrichment analysis of the whole transcriptome from MCF7 and MDA-MB-231 cells revealed that YAP1-induced genes were remarkably enriched in the *METTL14*-depleted groups [43] (Fig. 3i), and most of these genes were upregulated by *METTL14* depletion (Fig. 3j). These data suggest that *YAP1* is likely to be regulated by m^6^A.

### METTL14 regulates *YAP1* expression independently of the Hippo signaling

**Fig. 4:**
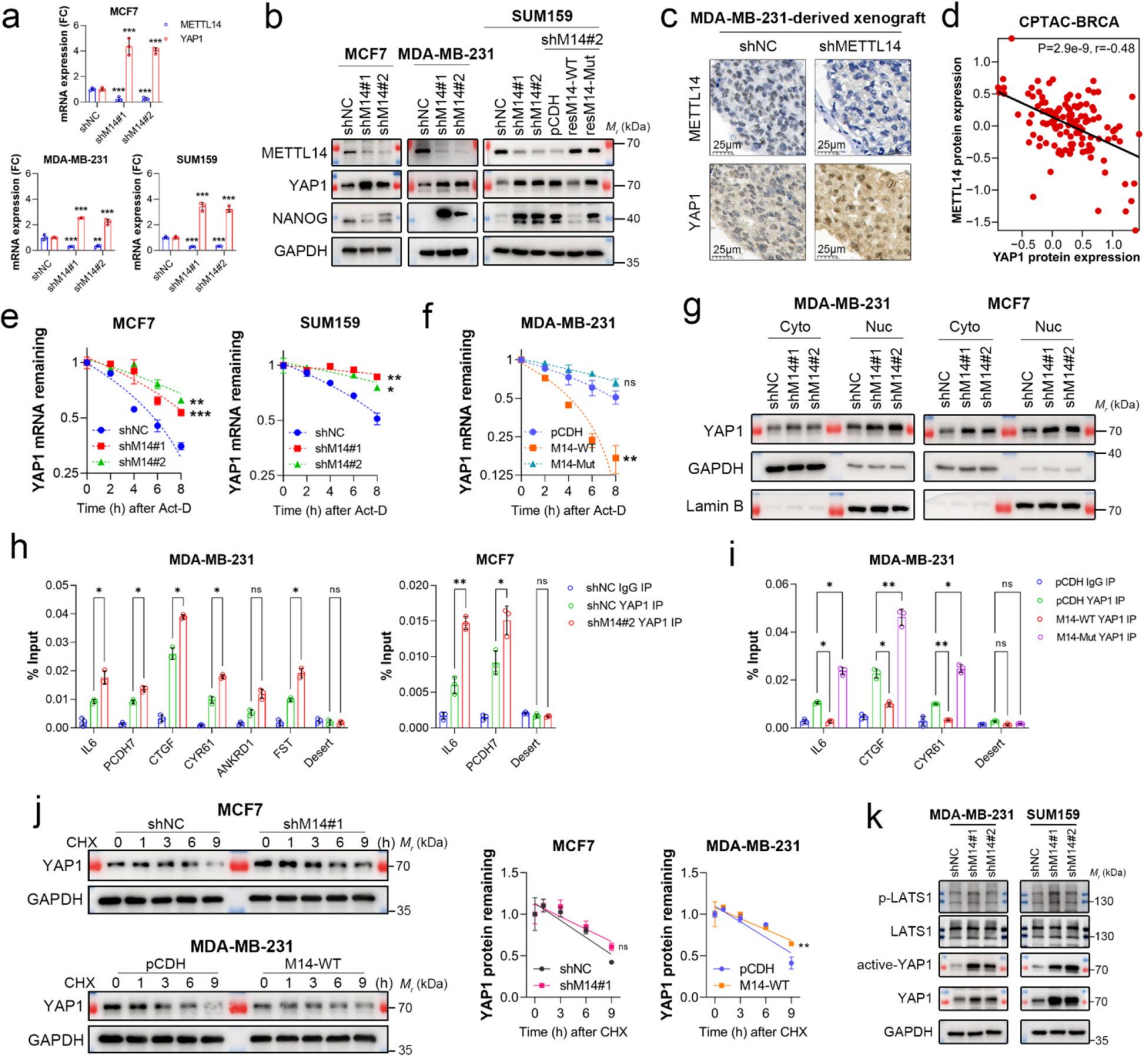
METTL14 regulates YAP1 expression independently of the Hippo signaling. **a** The mRNA expression of *YAP1* and *METTL14* in breast cancer (BC) cell lines with or without *METTL14* depletion was detected using qPCR. **b** Protein expression of METTL14, YAP1, and NANOG in BC cells with or without *METTL14* depletion or rescue of WT *METTL14* or the inactive mutant was determined by immunoblotting. GAPDH was used as the loading control. **c** Representative images of immunohistochemistry (IHC) staining for METTL14 and YAP1 in MDA-MB-231-derived xenografts. Scale bars: 25 μm. **d** Correlation between METTL14 and YAP1 protein expression in breast tumors from the Clinical Proteomic Tumor Analysis Consortium (CPTAC) dataset was determined using Pearson’s correlation coefficient (*n* = 134). **e, f** BC cell lines with or without *METTL14* depletion or overexpression of WT *METTL14* or the inactive mutant were treated with 5 μg/mL Act.D for the indicated time, and the stability of YAP1 mRNA was detected using qPCR. **g** Nuclear and cytoplasmic protein expression of YAP1 in BC cells with or without *METTL14* depletion was determined by immunoblotting. GAPDH was used as a loading control for cytoplasmic proteins, while Lamin B was used as a loading control for nuclear proteins. **h, i** YAP1 enrichment at the promoter of YAP1-induced target genes was determined using ChIP-qPCR analysis. **j** BC cell lines with or without *METTL14* depletion, overexpression of WT *METTL14*, or the inactive mutant were treated with 5 μg/mL CHX for the indicated time, and the stability of YAP1 was determined by immunoblotting. GAPDH was used as the loading control. **k** Protein expression of p-LATS1, LATS1, active YAP1, and YAP1 in TNBC cells with or without *METTL14* depletion was determined by immunoblotting. GAPDH was used as the loading control. Data are shown as the mean ± SD for a representative of three independent experiments performed in triplicate. *P < 0.05; **P < 0.01; ***P < 0.001; ns, nonsignificant

Consistent with the RNA-seq data, depletion of *METTL14* expression caused upregulation of *YAP1* mRNA and protein (Fig. 4a and b), and this upregulation was abolished by reconstituted expression of shRNA-resistant wild-type *METTL14*, but not the inactive mutant, in SUM159 cells (Fig. 4b). Results from six breast cancer cell lines transfected with *METTL14* siRNAs also showed a consistent upregulation of YAP1 protein (Suppl. Figure 4a). In addition, IHC staining of MDA-MB-231-derived xenografts and analysis of the CPTAC dataset demonstrated a negative correlation between METTL14 and YAP1 (Fig. 4c and d). *METTL14* knockdown had no significant effect on the other m^6^A regulators (Suppl. Figure 4b).

Given that MTTL14 is known to methylate mRNA, we reasoned that the increased *YAP1* expression in *METTL14-*depleted cells most likely resulted from improved mRNA stability. To determine if this was true, we treated breast cancer cells with actinomycin D (Act.D) and examined the decay rate of *YAP1* mRNA. The results of qPCR showed that the deletion of *METTL14* significantly prolonged the decay time of *YAP1* mRNA (Fig. 4e), whereas overexpression of the wild-type, but not the inactive *METTL14* mutant, accelerated the decay (Fig. 4f) and decreased *YAP1* expression level (Suppl. Figure 4c and d). In addition, we constructed a luciferase reporter plasmid by placing the promoter region (-800 to +100) of *YAP1* into the *pGL3-Basic* vector. Through luciferase reporter assay, we found that *METTL14* depletion slightly inhibited *YAP1* promoter activity (Suppl. Figure 4e), suggesting that METTL14 did not promote *YAP1* transcription. Additionally, *METTL14* depletion did not affect the distribution of *YAP1* mRNA in the nucleoplasm (Suppl. Figure 4f). However, it cannot be excluded that the translational efficiency of *YAP1* mRNA was promoted by *METTL14* depletion [44]. To determine the effect of METTL14 on *YAP1* mRNA translation, we constructed a *pmirGLO* reporter with m^6^A-modified regions (exon 10 and 3’UTR) of *YAP1* [29]. Luciferase reporter assay and qPCR of the luciferase gene showed that *METTL14* depletion slightly decreased the translational efficiency of *YAP1* mRNA (Suppl. Figure 4 g). These results suggest that *METTL14* depletion upregulates *YAP1* expression by stabilizing its mRNA.

We next asked if *METTL14* depletion-induced YAP1 is functioning and thus examined the transactivity of YAP1. As shown in Fig. 4g, both cytoplasmic and nuclear fractions of YAP1 increased in *METTL14-*depleted cells. More importantly, not only did nuclear translocation increase upon *METTL14* depletion, but also its transcriptional activity, as chromatin immunoprecipitation (ChIP) analysis showed that YAP1 was significantly enriched at the promoters of target genes [43], such as *IL6*, *CTGF*, *CYR61*, *PXCDH7*, and *FST* (Fig. 4h), which is consistent with the increased expression of these genes identified by RNA-seq (Suppl. Data 1). In contrast, overexpression of wild-type *METTL14* led to a significant reduction in YAP1 occupancy at these genes; however, the inactive *METTL14* mutant failed to generate similar results (Fig. 4i).

The Hippo pathway is the most important signal regulating YAP1 protein expression. Kinases in this pathway are often lost or inactivated in TNBC, leading to hyperactivated YAP1 signaling (Suppl. Figure 4 h) [19]. However, we found that the protein stability of YAP1 was not affected by *METTL14* depletion (Fig. 4j). *METTL14* depletion resulted in an increase in both total YAP1 and active YAP1 expression without influencing the activity of LATS1 (Fig. 4k), suggesting that the role of METTL14-mediated m^6^A modification in modulating YAP1 expression is independent of Hippo signaling.

### The m^6^A-modified 3’UTR triggers YTHDF2-dependent ***YAP1*** mRNA decay

**Fig. 5:**
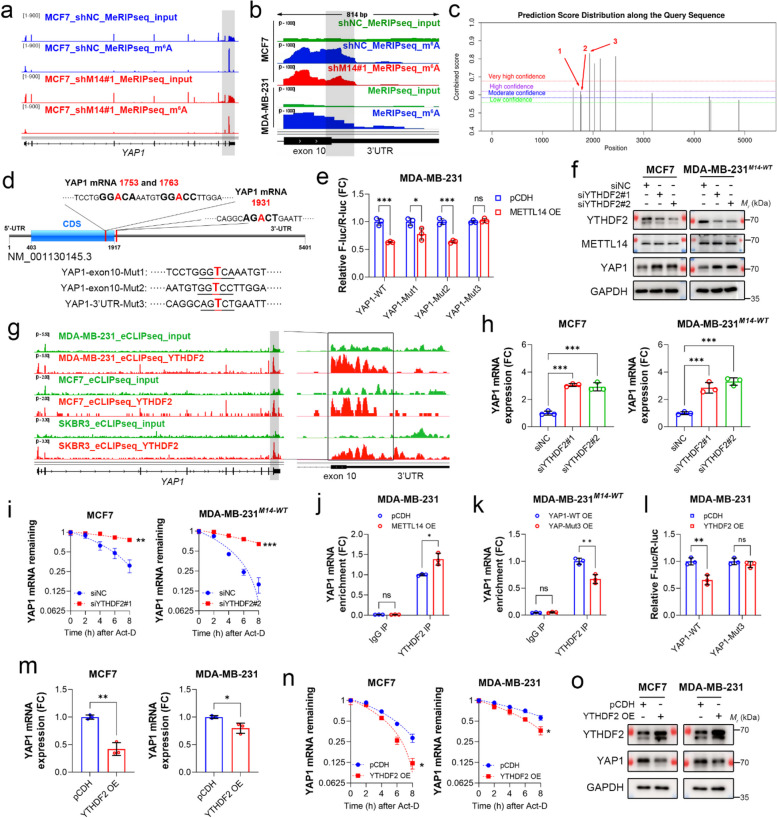
The m^6^A-modified 3’UTR triggers YTHDF2-dependent *YAP1* mRNA decay. **a** IGV shows the m^6^A signal in *YAP1* of MCF7 cells with or without *METTL14* depletion. **b** IGV shows the specific m^6^A peaks of *YAP1* in MCF7 and MDA-MB-231 cells. The gray square indicates the m^6^A peak downregulated by *METTL14* loss. **c** Mapping of m^6^A modification sites on *YAP1* mRNA by SRAMP. Red arrows indicate m^6^A sites identified by MeRIP sequencing data. **d** Scheme shows m^6^A motif positions within *YAP1* mRNA and their artificial mutations for luciferase reporter assays**. e** The *pmirGLO* reporter with WT or mutated exon 10–3’UTR region of *YAP1* was transfected into MDA-MB-231 cells with or without *METTL14* overexpression for 24 h, and the relative F-luc/R-luc ratio was determined using luciferase reporter assays. **f** Protein expression of YTHDF2, METTL14, and YAP1 in MCF7 and *METTL14*-overexpressing MDA-MB-231 cells, with or without *YTHDF2* knockdown, was detected by immunoblotting. GAPDH was used as the loading control. **g** IGV shows the YTHDF2 binding signals in *YAP1* of MDA-MB-231, MCF7, and SK-BR3 cells from the GSE137258 dataset. The square indicates the YTHDF2 binding peaks overlapped with the specific m^6^A peaks. **h** The mRNA expression of *YAP1* in MCF7 and *METTL14*-overexpressing MDA-MB-231 cells, with or without *YTHDF2* knockdown, was detected using qPCR. **i** MCF7 and *METTL14*-overexpressing MDA-MB-231 cells with or without *YTHDF2* knockdown were treated with 5 μg/mL Act.D for the indicated time. The stability of *YAP1* mRNA was determined by qPCR. **j** RIP was performed using anti-IgG control or anti-YTHDF2 antibodies in MDA-MB-231 cells with or without *METTL14* overexpression, followed by qPCR analysis of *YAP1* mRNA. **k** RIP was performed with anti-IgG control or anti-YTHDF2 antibodies in *METTL14*-overexpressing MDA-MB-231 cells with WT or mutated *YAP1* overexpression, followed by qPCR analysis of *YAP1* mRNA. **l** The *pmirGLO* reporter with WT or mutated exon10-3’UTR region of *YAP1* was transfected into MDA-MB-231 cells with or without *YTHDF2* overexpression for 24 h, and the relative F-luc/R-luc ratio was determined using a dual-luciferase reporter assay. **m** The mRNA expression of *YAP1* in MCF7 and MDA-MB-231 cells with or without *YTHDF2* overexpression was detected by qPCR. **n** MCF7 and MDA-MB-231 cells with or without *YTHDF2* overexpression were treated with 5 μg/mL Act.D for the indicated time. The stability of *YAP1* mRNA was determined by qPCR. **o** Protein expression of YTHDF2 and YAP1 in MCF7 and MDA-MB-231 cells with or without *YTHDF2* overexpression was detected using immunoblotting. GAPDH was used as the loading control. Data are shown as the mean ± SD for a representative of three independent experiments performed in triplicate. *P < 0.05; **P < 0.01; ***P < 0.001; ns, nonsignificant

We next sought to investigate the exact m^6^A site and reader that determines the stability of *YAP1* mRNA. Indeed, MeRIP sequencing data from MCF7 and MDA-MB-231 cells showed a specific m^6^A signal around the stop codon of *YAP1* (Fig. 5a and Suppl. Figure 5a). In contrast, no m^6^A signal was detected on the *NANOG* gene (Suppl. Figure 5b). Notably, there was a loss of m^6^A peak on the 3’UTR of mRNA, relative to the m^6^A peak on exon 10 in MDA-MB-231 cells, which is distinct from MCF7 shNC cells but similar to *METTL14*-depleted cells (Fig. 5b). Also, *YAP1* mRNA expression and stability in MDA-MB-231 cells were significantly higher than those in MCF7 cells (Suppl. Figure 5c and d), implying that this loss might play a role in determining *YAP1* expression. We next identified three potentially methylated adenosine bases within the specific m^6^A peak using a sequence-based site predictor [45] (Fig. 5c and Suppl. Data 3). To determine the effects of these m^6^A sites on *YAP1* expression, we constructed a luciferase gene reporter with the exon 10 and 3’UTR regions of *YAP1* containing wild-type or mutated m^6^A motifs (Fig. 5d). The reporter assay showed that *METTL14* overexpression significantly reduced luciferase activity with the wild-type, Mut1, and Mut2 fragments, whereas the Mut3 fragment conferred resistance to *METTL14* overexpression (Fig. 5e). These findings suggest that m^6^A-modified 3’UTR suppresses *YAP1* expression.

YT521-B homology (YTH) domain-containing family proteins, including YTHDF1, YTHDF2, and YTHDF3, are m^6^A readers with the ability to degrade mRNA. We speculated that these readers regulate *YAP1* expression by recognizing m^6^A marks. As expected, *YTHDF2* depletion notably increased YAP1 protein expression in MCF7 and *METTL14*-overexpressing MDA-MB-231 cells, without affecting METTL14 (Fig. 5f and Suppl. Figure 5e). Analysis of YTHDF2 cross-linking and immunoprecipitation datasets across multiple breast cancer cell lines showed that the binding signals of YTHDF2 covered the m^6^A-specific peak around the stop codon of *YAP1* [46] (Fig. 5g). Consistently, *YTHDF2* depletion led to a robust increase in the expression and stability of *YAP1* mRNA (Fig. 5h and 5i). Furthermore, *METTL14* overexpression increased YTHDF2 binding to *YAP1* mRNA (Fig. 5j), whereas overexpression of mutated *YAP1* decreased this binding (Fig. 5k). *YTHDF2* overexpression reduced luciferase activity driven by the wild-type exon 10–3’UTR fragment, while the mutated fragment rendered luciferase activity resistant to overexpression (Fig. 5l). In addition, *YTHDF2* overexpression reduced the expression and stability of *YAP1* mRNA (Fig. 5m and 5n) and led to a decrease in YAP1 protein (Fig. 5o). These results suggest that YTHDF2 is responsible for degrading *YAP1* mRNA by recognizing m^6^A-modified 3’UTR.

### YAP1 underlies the m^6^A-regulated stemness in TNBC

**Fig. 6:**
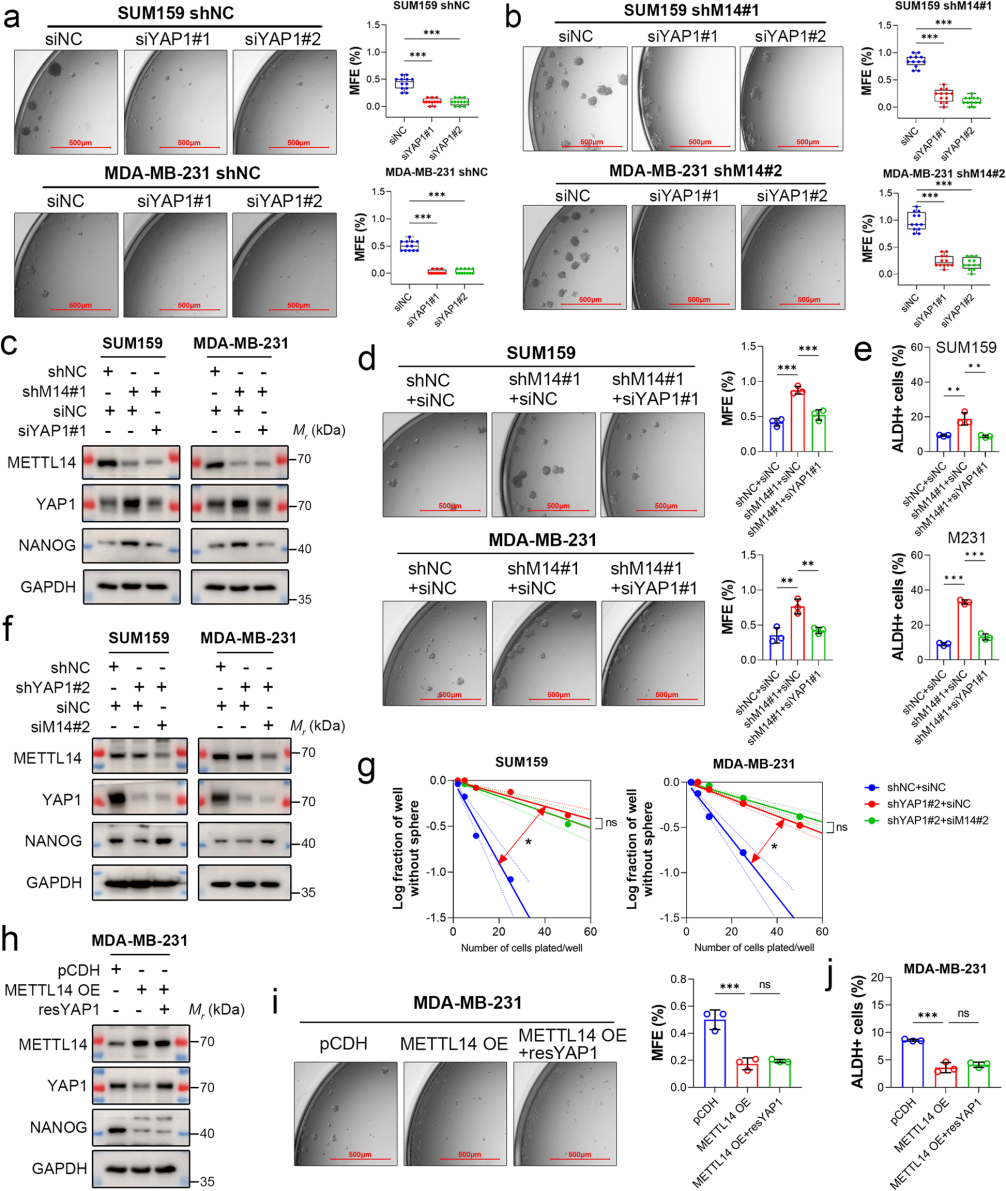
YAP1 underlies the m^6^A-regulated stemness in TNBC. **a** Representative images of the primary mammospheres of SUM159 and MDA-MB-231 shNC cells with or without *YAP1* knockdown. Scale bar: 500 μm. Data are shown as the mean ± SD of twelve replicate samples. **b** Representative images of primary mammospheres of *METTL14*-depleted SUM159 and MDA-MB-231 cells with or without *YAP1* knockdown. Scale bar: 500 μm. Data are shown as the mean ± SD of twelve replicate samples. **c** Protein expression of METTL14, YAP1, and NANOG in *METTL14*-depleted TNBC cells with or without *YAP1* knockdown was determined by immunoblotting. GAPDH was used as the loading control. **d** Representative images of the primary mammospheres of *METTL14*-depleted TNBC cell lines with or without *YAP1* knockdown. Scale bar: 500 μm. **e** Flow cytometric analysis of ALDH^+^ cells in *METTL14*-depleted TNBC cell lines with or without YAP1 knockdown. **f** Protein expression of METTL14, YAP1, and NANOG in *YAP1*-depleted TNBC cells with or without *METTL14* knockdown was determined by immunoblotting. GAPDH was used as the loading control. **g** The stemness of *YAP1*-depleted TNBC cells with or without *METTL14* knockdown was detected using in vitro limiting dilution assays. **h** Protein expression of METTL14, YAP1, and NANOG in *METTL14*-overexpressed MDA-MB-231 cells with or without ectopic *YAP1* expression was determined by immunoblotting. GAPDH was used as the loading control. **i** Representative images of primary mammospheres of *METTL14*-overexpressing and control MDA-MB-231 cells with or without ectopic *YAP1* expression. Scale bar: 500 μm. **j** Flow cytometric analysis of ALDH^+^ cells in *METTL14*-overexpressing and control MDA-MB-231 cells with or without ectopic *YAP1* expression. Unless otherwise stated, data are presented as the mean ± SD for a representative of three independent experiments performed in triplicate. *P < 0.05; **P < 0.01; ***P < 0.001; ns, nonsignificant

YAP1 is a specific regulator of stem-like properties in basal-like breast cancer cells [43]. *YAP1* knockdown resulted in decreased NANOG protein level (Suppl. Figure 6) and impaired the sphere-forming ability of MDA-MB-231 and SUM159 cells with or without *METTL14* depletion (Fig. 6a and b). To determine the essentiality of YAP1 as an m^6^A-modified target in maintaining TNBC stemness, we used siRNAs to knock down *YAP1* expression in *METTL14*-depleted TNBC cells to a level comparable to that in shNC cells (Fig. 6c). The knockdown reduced NANOG protein expression (Fig. 6c) and stemness of *METTL14*-depleted TNBC cells to levels comparable to shNC cells (Fig. 6d and e). To examine whether the essentiality is a consequence of the transforming property of stable *METTL14* depletion, we generated stable SUM159 and MDA-MB-231 cell lines expressing shRNAs targeting *YAP1* (sh*YAP1*). Consistent with *YAP1* knockdown by siRNA transfection (Suppl. Figure 6), stable *YAP1* depletion modestly decreased the protein expression of NANOG in SUM159 and MDA-MB-231 cells (Fig. 6f) and disrupted their potential to form spheres (Fig. 6g). Knockdown of *METTL14* in *YAP1*-depleted SUM159 and MDA-MB-231 cells failed to improve their sphere-forming capability (Fig. 6g), though an increase in NANOG protein level was observed (Fig. 6f). These results indicate that *YAP1* is essential for METTL14-regulated stemness in TNBC.

We next investigated whether restoring *YAP1* was sufficient to reverse METTL14-induced inhibition of stemness by transiently expressing *YAP1* in *METTL14*-overexpressing MDA-MB-231 cells. Immunoblotting analysis showed that ectopic expression of *YAP1* failed to rescue the protein expression of NANOG (Fig. 6h), suggesting that NANOG is not a target of YAP1. Since NANOG is not m^6^A-modified either, it is likely a marker associated with METTL14-regulated TNBC stemness. Furthermore, ectopic expression of *YAP1* failed to restore the stemness of *METTL14*-overexpressing MDA-MB-231 cells (Fig. 6i and j). These findings indicate that YAP1 is necessary, but not sufficient, for m^6^A-regulated TNBC stemness.

### The expression of *METTL14* is transcriptionally suppressed by LSD1

**Fig. 7:**
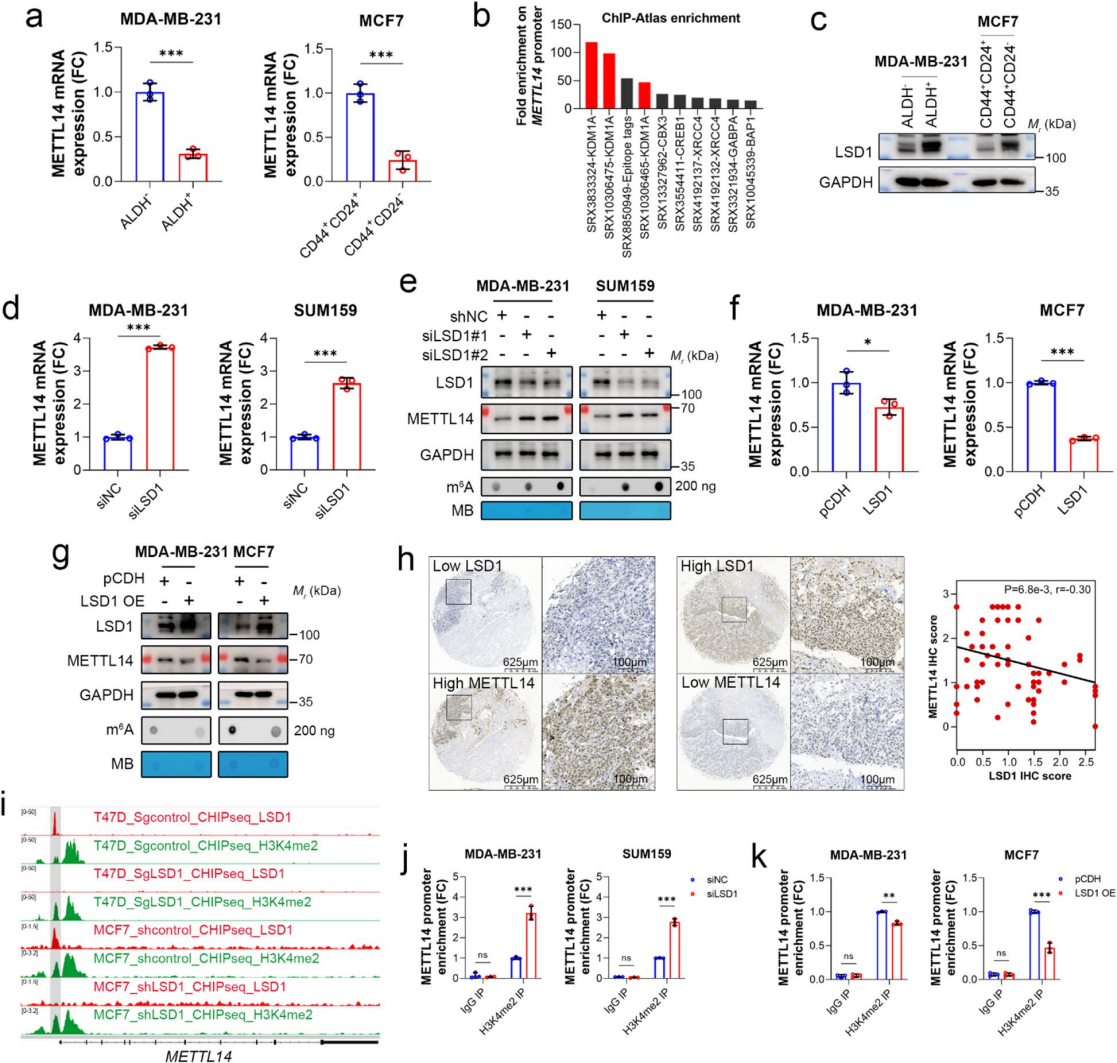
The expression of *METTL14* is transcriptionally suppressed by LSD1. **a** The mRNA expression of *METTL14* in breast cancer stem cells (BCSCs) and non-BCSCs was detected using qPCR. **b** Analysis of transcription and chromatin regulators of *METTL14* in breast cells using the CHIP-Atlas data-mining suite. **c** Protein expression of LSD1 and METTL14 in BCSCs and non-BCSCs was determined using immunoblotting. GAPDH was used as the loading control. **d** The mRNA expression of *METTL14* in MDA-MB-231 and SUM159 cells with or without *LSD1* knockdown was detected by qPCR. **e** Protein expression of LSD1 and METTL14 in MDA-MB-231 and SUM159 cells with or without *LSD1* knockdown was determined by immunoblotting. GAPDH was used as the loading control. The m^6^A levels of global mRNA in these cells were determined using dot blotting. Methylene blue (MB) staining was used as the loading control. **f** The mRNA expression of *METTL14* in MDA-MB-231 and MCF7 cells with or without *LSD1* overexpression was detected by qPCR. **g** Protein expression of LSD1 and METTL14 in MDA-MB-231 and MCF7 cells with or without *LSD1* overexpression was detected by immunoblotting. GAPDH was used as the loading control. Global m^6^A levels of mRNA in these cells were determined using dot blotting. MB staining was used as a loading control. **h** Representative images of immunohistochemical (IHC) staining for METTL14 and LSD1 in human TNBC tissues (*n* = 80). Scale bars: 625 and 100 μm. IHC staining score was calculated, and the correlation was determined using the Pearson correlation coefficient. **i** The upper IGV visualization shows LSD1 and H3K4me2 signals on the *METTL14* promoter of T47D cells with or without *LSD1* knockout from the GSE168644 dataset. The lower IGV visualization shows LSD1 and H3K4me2 signals at the *METTL14* promoter of MCF7 cells with or without *LSD1* knockdown from the GSE112230 dataset. **j** The binding abundance of H3K4me2 at the *METTL14* promoter in MDA-MB-231 and SUM159 cells with or without *LSD1* knockdown was determined by ChIP-qPCR. **k** The binding abundance of H3K4me2 at the *METTL14* promoter in MDA-MB-231 and MCF7 cells with or without *LSD1* overexpression was determined by ChIP-qPCR. Data are presented as the mean ± SD for a representative of three independent experiments performed in triplicate. *P < 0.05; **P < 0.01; ***P < 0.001; ns, nonsignificant

*METTL14* expression was significantly lower in TNBC than in luminal cancer (Suppl. Figure 1b). Similar results were also observed in BCSCs (Fig. 7a). Hence, we speculated that low expression of *METTL14* might result from transcriptional repression. To identify the factors that govern the transcription of *METTL14*, we consulted the CHIP-seq data accessible in the CHIP-Atlas data-mining suite and found that lysine-specific demethylase 1 (LSD1, also known as KDM1A) displayed the highest binding abundance at the promoter region of *METTL14* [47] (Fig. 7b and Suppl. Data 4). In contrast to the expression pattern of *METTL14* (Fig. 1f), LSD1 was highly expressed in ALDH^+^ or CD44^+^CD24^−^ BCSCs (Fig. 7c). Furthermore, *LSD1* knockdown by two different siRNAs resulted in a marked elevation of *METTL14* mRNA in MDA-MB-231 and SUM159 cells (Fig. 7d). METTL14, as well as global m^6^A levels in MDA-MB-231 and SUM159 cells, were also significantly upregulated by *LSD1* knockdown (Fig. 7e). However, the other m^6^A regulators were not affected by *LSD1* depletion (Suppl. Figure 7a). In addition, *LSD1* overexpression in MDA-MB-231 and MCF7 cell lines effectively reduced the mRNA and protein expression of *METTL14* as well as global m^6^A modification (Fig. 7f and g). Immunostaining of LSD1 in human TNBC tissues showed that the intratumoral protein level of LSD1 was inversely correlated with that of METTL14 (R = -0.3, *P* = 0.0068) (Fig. 7h). These results suggest that the loss of *METTL14* in TNBC and BCSCs may be due to the high expression of *LSD1*.

LSD1 is a well-known transcription repressor that demethylates mono- or di-methylated histone H3 lysine 4 (H3K4me1/2) [48]. Analysis of the CHIP-seq datasets (GSE168644 and GSE112230) derived from various breast cancer cell lines showed that LSD1 and H3K4me2 specifically bind to the transcription start site of *METTL14* (Fig. 7i), whereas H3K4me1 was not found (Suppl. Figure 7b). Notably, when *LSD1* was knocked down or knocked out in MCF7 and T47D cell lines, respectively, there was a consistent increase in the abundance of H3K4me2 at the *METTL14* promoter (Fig. 7i), suggesting that LSD1 may control *METTL14* expression by regulating H3K4me2 levels. To further confirm the role of LSD1, we performed CHIP-qPCR analysis in TNBC cells using specific primers targeting the H3K4me2 binding region within the *METTL14* promoter. The results showed that *LSD1* depletion led to a substantial increase in the abundance of H3K4me2 at the *METTL14* promoter (Fig. 7j), whereas overexpression of *LSD1* resulted in a decrease (Fig. 7k). These findings provide compelling evidence that LSD1 acts as a negative regulator of *METTL14* expression in breast cancer.

### Loss of *METTL14* is critical for LSD1-driven stemness in TNBC

**Fig. 8:**
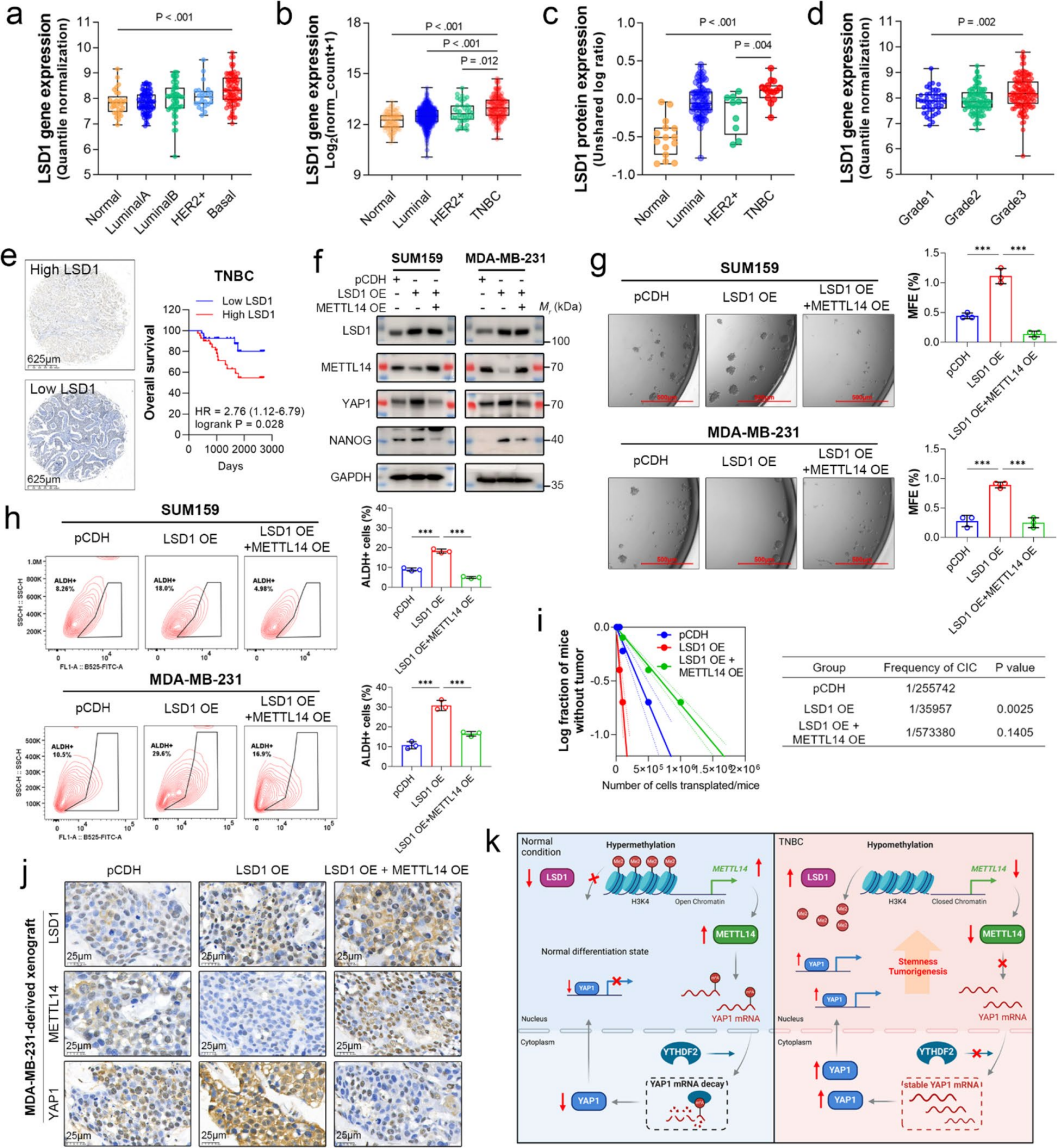
Loss of *METTL14* is critical for LSD1-driven stemness in TNBC. **a** Expression of *LSD1* in normal adjacent (*n* = 29), luminal A (*n* = 89), luminal B (*n* = 49), HER2^+^ (*n* = 24), and basal tumor (*n* = 75) tissues from the GSE21635 cohort. Data are shown as the mean ± SD of the normalized microarray expression. **b** Expression of *LSD1* in normal adjacent (*n* = 113), non-TNBC (*n* = 860), HER2^+^ (*n* = 38), and TNBC (*n* = 192) tissues from The Cancer Genome Atlas (TCGA) cohort. Data are presented as the mean ± SD of the normalized read counts. **c** Protein expression of LSD1 in normal adjacent (*n* = 15), luminal (*n* = 75), HER2^+^ (*n* = 9), and TNBC (*n* = 15) tissues was obtained from the Clinical Proteomic Tumor Analysis Consortium (CPTAC). Data are shown as the mean ± SD of normalized protein expression. **d** Expression of *LSD1* in Scarff-Bloom-Richardson grade1 (*n* = 45), grade2 (*n* = 89), and grade3 (*n* = 125) tissues from the GSE21635 cohort. Data are shown as the mean ± SD of the normalized microarray expression. **e** Representative images of immunohistochemical (IHC) staining for LSD1 in human TNBC tissues. Scale bars: 625 μm. Survival analysis of TNBC patients (*n* = 80, from the tissue microarray) stratified by LSD1 IHC score (median) was conducted using the log-rank test. **f** Protein expression of LSD1, METTL14, YAP1, and NANOG in TNBC cells with or without *LSD1* overexpression and *METTL14* rescue was detected by immunoblotting. GAPDH was used as the loading control. **g** Representative images of the primary mammospheres of TNBC cells with or without *LSD1* overexpression and *METTL14* rescue. Scale bar: 500 μm. Data are shown as the mean ± SD for a representative of three independent experiments performed in triplicate. ***P < 0.001. **h** Flow cytometric analysis of ALDH^+^ cell population in TNBC cells with or without *LSD1* overexpression and *METTL14* rescue. Data are shown as the mean ± SD for a representative of three independent experiments performed in triplicate. ***P < 0.001. **i** In vivo limiting dilution assay shows the frequency of cancer initiation cells (CICs) in MDA-MB-231 cells with or without *LSD1* overexpression and *METTL14* rescue (*n* = 5 per group). Quantification of CIC frequency was performed using L-Calc software. **j** Representative images of IHC staining for LSD1, METTL14, and YAP1 in MDA-MB-231-derived xenografts. Scale bars: 25 μm. **k** LSD1 exhibits elevated expression levels in TNBC and functions to repress the transcription of *METTL14* by demethylating H3K4me2 at the promoter region. This demethylation leads to a decrease in *METTL14* expression and subsequent reduction in global m^6^A levels. Loss of m^6^A prevents the recognition and degradation of *YAP1* mRNA by YTHDF2, thereby sustaining the hyperactivation of YAP1 signaling, which is a key factor contributing to the pronounced stemness observed in TNBC

LSD1 plays a crucial role in the growth and metastasis of breast cancer [49, 50]. Analysis of the GSE21653 and TCGA breast cancer cohort showed that *LSD1* was highly expressed in breast cancer with basal-like traits (Fig. 8a and b). Data from the CPTAC datasets also showed that TNBC exhibited much higher LSD1 protein expression than normal breast and luminal cancer tissues (Fig. 8c). Also, high expression of LSD1 was associated with a higher Scarff-Bloom-Richardson grade in breast cancer (Fig. 8d). The high intratumoral protein level of LSD1 correlated with a shorter OS time in patients with TNBC (Fig. 8e). These results support the association of *LSD1* with a poorly differentiated state and worse prognosis in breast cancer.

We next asked whether *METTL14* was critical for LSD1-driven stemness. To this end, we overexpressed *LSD1* with or without rescuing *METTL14* expression in SUM159 and MDA-MB-231 cells. As expected, *LSD1* overexpression reduced the endogenous protein expression of METTL14 but increased that of YAP1 and NANOG (Fig. 8f). Rescue of *METTL14* in these cells abolished the inducible effects of LSD1 on YAP1 and NANOG (Fig. 8f). Mammosphere formation assays showed that the sphere-forming capacity of TNBC cells was significantly enhanced by *LSD1* overexpression; however, co-expression with *METTL14* eliminated this effect (Fig. 8g). Flow cytometric analysis of ALDH^+^ cells also demonstrated that *METTL14* rescue compromised the inducible effect of LSD1 on stemness (Fig. 8h). Consistently, in vivo LDA showed that the frequency of CIC in *METTL14* rescue group was remarkably decreased compared to *LSD1* overexpression alone (Fig. 8i). IHC staining of xenografts derived from these cells confirmed the essential role of *METTL14* loss in LSD1-induced YAP1 expression (Fig. 8j). These results suggest that the function of LSD1 in promoting TNBC stemness is largely mediated by repressing *METTL14* expression.

## Discussion

We show that the loss of *METTL14*, resulting from LSD1-mediated histone demethylation, favors the stemness of TNBC by interfering with YTHDF2-mediated degradation of *YAP1* transcript (Fig. 8k).

The role of METLL14 in cancer is multi-faceted. For example, in leukemia, METTL14 was shown to promote stemness by inducing *MYC* and *MYB* expression [51]. Similarly, METTL14 promotes tumorigenicity and chemoresistance in osteosarcoma by inducing *MN1* expression [52]. In contrast to its oncogenic effect, METTL14 was found to trigger ferroptosis in endocervical cancer [53], impede the tumor growth of ocular melanoma [54], and promote genome-wide repair to prevent skin cancer [55], acting as a tumor suppressor. The diverse functions of METTL14 in these cancers may be attributed to tissue-specific expression or regulation [30]. However, the role of METTL14 in breast cancer remains controversial. While one study showed that suppressing *METTL14* with siRNA impaired colony formation and invasion of TNBC cells [42], two recent clinical investigations indicated that low expression of *METTL14* in breast cancer tissue is associated with shorter survival time of patients and is an independent unfavorable indicator for TNBC [32, 33]. Despite the fact that the cohorts in these studies are derived from various public datasets and regions and underwent distinct prognostic evaluations, the collective findings indicate that the loss of *METTL14* is associated with an unfavorable prognosis in TNBC. Our analysis of proteomic data from breast cancer, sourced from CPTAC and TCGA, demonstrated that METTL14 is consistently and significantly downregulated in TNBC, aligning with the observed decrease in m^6^A levels. In addition, it was reported that METTL14 can induce H3K27me3 demethylation by enlisting KDM6B at the chromatin level [56]. This function is not dependent on methyltransferase activity but plays a role in the differentiation of mouse embryonic stem cells. Our results showed that the expression of *METTL14* mutant with abrogated methyltransferase activity robustly promoted sphere formation, likely due to dominant-negative effects, suggesting a suppressive role of m^6^A in TNBC stemness.

The hyperactivation of YAP1 in TNBC is considered due to the silencing of Hippo signaling. For instance, O-GlcNAc glycosylation of LATS2 blocks its phosphorylation by MST [19]. Similarly, the Cullin 2 E3 ubiquitin ligase subunit PRAMEF2 promotes the degradation of LATS1 by catalyzing its polyubiquitination [20]. Furthermore, non-coding RNAs can also inhibit LATS1 through various mechanisms [21, 22]. In this study, we identified YAP1 as an m^6^A-regulated target from both MCF7 and MDA-MB-231 cells. One of the primary reasons for selecting MCF7 for sequencing is its comparatively higher expression of METTL14 and m^6^A levels relative to MDA-MB-231, rendering it more susceptible to *METTL14* knockdown, which facilitates the screening of DEGs associated with METTL14. While our initial focus was on the differences between luminal breast cancer and MSL TNBC, this does not mean that the role of METTL14 in determining YAP1 expression is opposite in these two breast cancer subtypes. *METTL14* knockdown in MCF7 cells also resulted in the upregulation of YAP1, mirroring the response observed in TNBC, suggesting that the underlying molecular mechanism is consistent across various breast cancer subtypes, irrespective of the Hippo pathway activity.

Notably, the responses of different breast cancer subtypes to YAP1 upregulation are quite different. A recent study has shown that high expression of YAP1 is detrimental to ER^+^ breast cancer [18]. Another investigation showed that an lncRNA promotes the progression of ER^+^ and HER2^+^ breast cancer by promoting METTL14-mediated m^6^A modification [57]. In alignment with these findings, our observations revealed that the knockdown of *METTL14* resulted in growth inhibition in MCF7 cells while having no substantial impact on the growth of TNBC (data not shown). Therefore, it appears that high levels of METTL14 expression may be unfavorable in subtypes other than TNBC owing to the downregulation of YAP1, which contrasts with the behavior observed in TNBC. It was documented that YAP1 can activate the transcription of ornithine decarboxylase 1 and trigger the polyamine-dependent translation of LSD1 [58]. Thus, our data and those of others suggest a feedback loop for maintaining cell stemness.

Although we demonstrated that YAP1 is necessary for m^6^A-regulated stemness of TNBC, rescue of *YAP1* is not sufficient to reverse the suppressive effects of METTL14 on stemness. This suggests that activation cues other than YAP1 are also required for m^6^A-regulated stemness. It is noteworthy that the overexpression of METTL14 also resulted in a reduction of NANOG, one of the Yamanaka factors, although this effect occurred independently of m^6^A modification. It is difficult to determine whether this change is a consequence of weakened stemness or due to the function of METTL14 at the chromatin level. Further exploration of the mechanisms by which NANOG cooperates with METTL14 to maintain breast cancer stemness is necessary to answer this question. Nevertheless, YAP1 is considered one of the therapeutic targets for TNBC [59]. A recent investigation employed YAP1 inhibitors in the treatment of TNBC, yielding encouraging results from both in vitro and in vivo models [60]. Thus, our findings offer additional biomarkers for identifying patients who may benefit from YAP1 inhibitors and propose alternative strategies for targeting YAP1.

LSD1 belongs to the flavin adenine dinucleotide-dependent monoamine oxidase family and is the first histone demethylase discovered. The main function of LSD1 is to reduce chromatin accessibility by demethylating H3K4me1/2 [48] and to promote the transcriptional activation of hormone receptors by demethylating H3K9me1/2 [61, 62]. In breast cancer, LSD1 is controlled by ubiquitin and is involved in the fine-tuning of gene expression programs associated with differentiation, similar to its function in embryonic stem cells [63]. For example, LSD1 is deubiquitinated and stabilized by USP28 and OTUD7B to support breast cancer stemness and metastasis [50, 64]. Furthermore, LSD1 was found to regulate immune-related genes, and inhibition of LSD1 improves the immunotherapy of TNBC [65]. Our results demonstrated that LSD1 can upregulate *YAP1* expression and promote stem-like properties in TNBC largely by repressing the transcription of *METTL14*, suggesting that the loss of *METTL14* is another mechanism critical for the function of LSD1 in determining the aggressiveness of cancer cells. Because LSD1 and METTL14 regulate gene expression at different levels via crosstalk, *METTL14* expression level may serve as an indicator for the efficacy of LSD1-based cancer therapy.

## Conclusion

This study indicates that LSD1 disrupts the transcription of *METTL14* in TNBC by demethylating histone at the promoter region, which subsequently diminishes global m^6^A modification. The reduction in m^6^A modification prevents YTHDF2-mediated degradation of *YAP1* transcript, resulting in hyperactivated YAP1 signaling that is essential for maintaining the stemness of TNBC. These results reveal an interplay of epigenetic modifications involved in the maintenance of TNBC stemness.

## Supplementary Information

Supplementary Material 1.

Supplementary Material 2.

Supplementary Material 3.

Supplementary Material 4.

Supplementary Material 5.

Supplementary Material 6.

## Data Availability

The MeRIP-seq and RNA-seq datasets pertaining to MCF7 can be accessed publicly in GEO under accession number GSE245282. Additional data are provided in the supplementary materials and can be obtained upon reasonable request from the corresponding author.

## Abbreviations

TNBC: Triple-negative breast cancer
m^6^A: N^6^-methyladenosine
MSL: Mesenchymal stem-like
METTL14: Methyltransferase-like 14
YAP1: Yes-associated protein 1
YTHDF2: YTH domain-containing family protein 2
LSD1: Lysine-specific demethylase 1
H3K4: Histone H3 lysine 4
BRCA: Breast cancer susceptibility gene
BCSC: Breast cancer stem cell
MST: Macrophage-stimulating protein
LATS: Large tumor suppressor kinase
RIP: RNA immunoprecipitation
ChIP: Chromatin immunoprecipitation
IHC: Immunohistochemistry
GESA: Gene set enrichment analysis
qRT-PCR: Quantitative real-time PCR
TCGA: The Cancer Genome Atlas
CPTAC: Clinical Proteomic Tumor Analysis Consortium
