# Supplementary material for: METTL14 suppresses the expression of YAP1 and the stemness of triple-negative breast cancer
Source: J Exp Clin Cancer Res. 2024 Nov 20;43:307. doi: 10.1186/s13046-024-03225-2 (PMC11577812; doi:10.1186/s13046-024-03225-2)
Supplement: Supplementary file 1 — Supplementary Material 1. [file 13046_2024_3225_MOESM1_ESM.docx]

**Supplementary Tables and Figures**

**METTL14 Suppresses the Expression of YAP1 and the Stemness of Triple-Negative Breast Cancer**

Xupeng Bai^*^, Jiarui Liu, Shujie Zhou, Lingzhi Wu, Xiaojie Feng, Pumin Zhang^*^

Suppl. Table 1. Target sequences for siRNA/shRNAs

Suppl. Table 2. Antibodies

Suppl. Table 3. Primers for qRT-PCR and ChIP-qPCR analysis

Suppl. Fig. 1: Loss of *METTL14* correlates with TNBC stemness

Suppl. Fig. 2: METTL14-mediated m^6^A modification suppresses the stem-like trait of TNBC

Suppl. Fig. 3: Identification of m^6^A-regulated transcripts involved in TNBC stemness

Suppl. Fig. 4: METTL14 regulates YAP1 expression independently of the Hippo signaling

Suppl. Fig. 5: The m^6^A-modified 3’UTR triggers YTHDF2-dependent *YAP1* mRNA decay

Suppl. Fig. 6: YAP1 underlies the m^6^A-regulated stemness in TNBC

Suppl. Fig. 7: The expression of *METTL14* is transcriptionally suppressed by LSD1

**Suppl. Table 1. Target sequences for siRNA/shRNAs**

| **Oligonucleotides** | **Species** | **Target sequences (5’→3’)** |
| --- | --- | --- |
| siNC/shNC | Human | CAACAAGATGAAGAGCACCAA |
| shMETTL14#1 | Human | CCATGTACTTACAAGCCGATA |
| shMETTL14#2 | Human | GCCGTGGACGAGAAAGAAATA |
| siMETTL14#1 | Human | CCATGTACTTACAAGCCGATA |
| siMETTL14#2 | Human | GCCGTGGACGAGAAAGAAATA |
| siMETTL14#3 | Human | GCTAATGTTGACATTGACTTA |
| siYAP1#1 | Human | GCCACCAAGCTAGATAAAGAA |
| siYAP1#2 | Human | CAGGTGATACTATCAACCAAA |
| shYAP1 | Human | GCCACCAAGCTAGATAAAGAA |
| siYTHDF1 | Human | GTTCGTTACATCAGAAGGATA |
| siYTHDF2#1 | Human | GCAGACTTGCAGTTTAAGTAT |
| siYTHDF2#2 | Human | GATGGATTAAACGATGATGAT |
| siYTHDF3 | Human | GCAAGGAAATAAAGTTTCAGT |
| siLSD1#1 | Human | GCCTAGACATTAAACTGAATA |
| siLSD1#2 | Human | CCACGAGTCAAACCTTTATTT |
| shLSD1 | Human | CCACGAGTCAAACCTTTATTT |

**Suppl. Table 2. Antibodies**

| **Antibodies** | **Sources** | **Catalog#** |
| --- | --- | --- |
| Rabbit anti-METTL3 | Cell Signaling Technology | 86132S |
| Rabbit anti-METTL14 | Cell Signaling Technology | 48699S |
| Rabbit anti-METTL16 | Proteintech | 19924-1-AP |
| Rabbit anti-WTAP | Sangon Biotech | D152385 |
| Rabbit anti-FTO | Proteintech | 27226-1-AP |
| Rabbit anti-ALKBH5 | Cell Signaling Technology | 80283S |
| Rabbit anti-m^6^A | Cell Signaling Technology | 56593S |
| Rabbit anti-YAP1 | Cell Signaling Technology | 14074S |
| Rabbit anti-LSD1 | Proteintech | 20813-1-AP |
| Rabbit anti-OCT4 | Abcam | ab200834 |
| Rabbit anti-NANOG | Abcam | ab109250 |
| Rabbit anti-MST1 | Proteintech | 22245-1-AP |
| Rabbit anti-p-LATS1 | Proteintech | 28998-1-AP |
| Rabbit anti-LATS1 | Proteintech | 17049-1-AP |
| Rabbit anti-YTHDF2 | Proteintech | 24744-1-AP |
| Rabbit anti-GAPDH | Sangon Biotech | D110016 |
| Rabbit anti-Lamin B | Proteintech | 12987-1-AP |
| Mouse anti H3K4me2 | Proteintech | 91321 |
| Anti-mouse secondary antibody | Cell Signaling Technology | 7076 |
| Anti-rabbit secondary antibody | Cell Signaling Technology | 7074 |
| CoraLite^®^ Plus 488-conjugated mouse anti-human CD49f | Proteintech | CL488-66906 |
| APC-conjugated mouse anti-human CD44 | Proteintech | APC-65063 |
| CoraLite^®^594-conjugated mouse anti-human CD24 | Proteintech | CL594-67627 |

**Suppl. Table 3. Primers for qRT-PCR and ChIP-qPCR**

| **Names** | **Application** | **Species** | **Primer sequences (5’→3’)** |
| --- | --- | --- | --- |
| YAP1-Fwd | qRT-PCR | Human | TAGCCCTGCGTAGCCAGTTA |
| YAP1-Rv | qRT-PCR | Human | TCATGCTTAGTCCACTGTCTGT |
| METTL14-Fwd | qRT-PCR | Human | GAACACAGAGCTTAAATCCCCA |
| METTL14-Rv | qRT-PCR | Human | TGTCAGCTAAACCTACATCCCTG |
| GAPDH-Fwd | qRT-PCR | Human | GGAGCGAGATCCCTCCAAAAT |
| GAPDH-Rv | qRT-PCR | Human | GGCTGTTGTCATACTTCTCATGG |
| F-luc-Fwd | qRT-PCR | Firefly | GCCATGAAGCGCTACGCCCTGG |
| F-luc-Rv | qRT-PCR | Firefly | TCTTGCTCACGAATACGACGGTGG |
| R-luc-Fwd | qRT-PCR | Renilla | TCAGTGGTGGGCTCGCTGCA |
| R-luc-Rv | qRT-PCR | Renilla | CTTTGGAAGGTTCAGCAGCTCG |
| PCDH7-Fwd | ChIP-qPCR | Human | CGCAACCATCCAAAGTCTG |
| PCDH7-Rv | ChIP-qPCR | Human | CCCAGAAAGCCACTCTGTTC |
| FST-Fwd | ChIP-qPCR | Human | TAAAGCACTCCGGATCTTGC |
| FST-Rv | ChIP-qPCR | Human | TGCGTGCTTTGTAAGTGTCC |
| CTGF-Fwd | ChIP-qPCR | Human | GGAGTGGTGCGAAGAGGATA |
| CTGF-Rv | ChIP-qPCR | Human | GCCAATGAGCTGAATGGAGT |
| IL6-Fwd | ChIP-qPCR | Human | CTGCAAGTTCCCACAGTTCA |
| IL6-Rv | ChIP-qPCR | Human | CCCACCTTCTTCAAAATCCA |
| CYR61-Fwd | ChIP-qPCR | Human | CAAGAATGCTTTGTGGTTGG |
| CYR61-Rv | ChIP-qPCR | Human | GGTGAATCAGACACCAGACG |
| ANKRD1-Fwd | ChIP-qPCR | Human | GGCACTTTTCTATGCAGTTGG |
| ANKRD1-Rv | ChIP-qPCR | Human | TTCTGGCAGCATATTTCAGC |
| Desert-Fwd | ChIP-qPCR | Human | GTTCATCCCAGCACCTGTCT |
| Desert-Rv | ChIP-qPCR | Human | GTGATGGACCTGGAGCCTAA |
| METTL14-Fwd | ChIP-qPCR | Human | ACTAGCCAAGGCCACTCGTCT |
| METTL14-Rv | ChIP-qPCR | Human | GCAGGTTGGGGTGGTCTGAG |

**
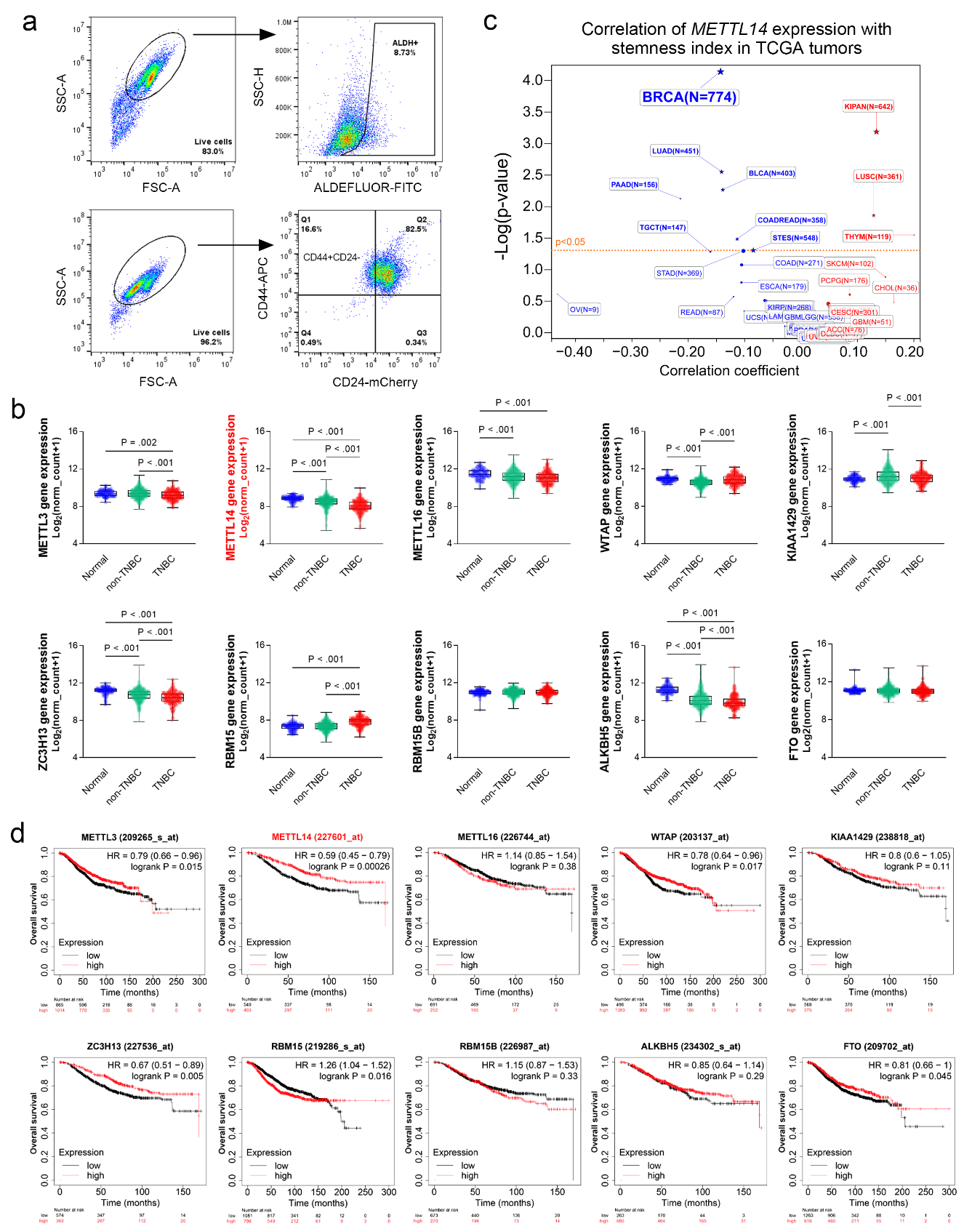
**

**Suppl. Fig. 1: Loss of *METTL14* correlates with TNBC stemness.** **a** Flow cytometry gating strategy for sorting ALDH^+^ or CD44^+^CD24^-^ breast cancer stem cells from various human breast cancer cell lines. **b** Gene expression of *METTL3, METTL14, METTL6, WTAP, KIAA1429, ZC3H13, RBM15, RBM15B, FTO,* and *ALKBH5* in normal adjacent (n=113), non-TNBC (n=898), and TNBC (n=194) tissues from the Cancer Genome Atlas (TCGA) cohort was compared using a two-tailed t-test. Data are presented as the mean ± SD of the normalized read counts. **c** Correlation analysis of *METTL14* gene expression with tumor stemness in TCGA pan-cancer dataset was performed using Pearson’s correlation test. **d** Survival curves of BC patients stratified by *METTL3, METTL14, METTL6, WTAP, KIAA1429, ZC3H13, RBM15, RBM15B, FTO,* and *ALKBH5* expression were plotted and analyzed using the KM plotter.

**
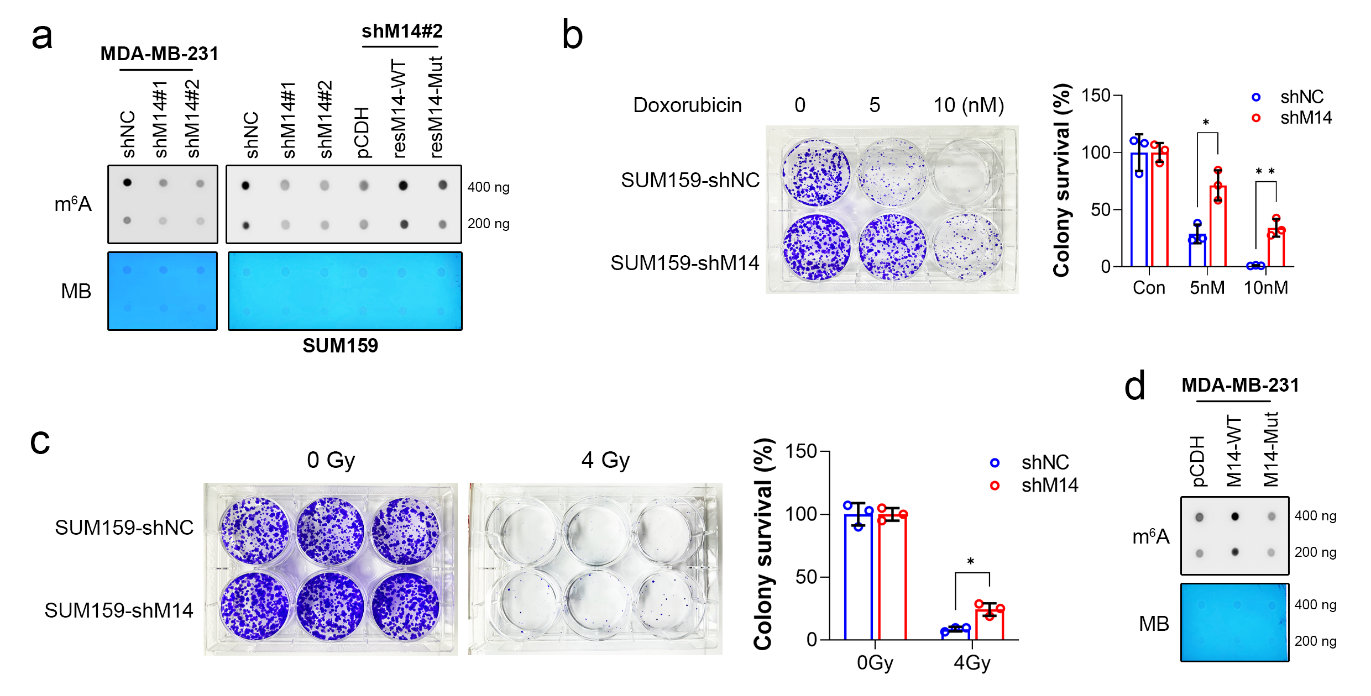
**

**Suppl. Fig. 2: METTL14-mediated m^6^A modification suppresses the stem-like trait of TNBC. a** The m^6^A level of global mRNA in MDA-MB-231 and SUM159 cells with or without *METTL14* depletion or rescue of wild-type (WT) or mutated *METTL14* was determined by dot blotting. Methylene blue (MB) staining was used as the loading control. **b** Representative images of colonies formed by SUM159 shNC and shM14 cells treated with doxorubicin (DOX) for 48 h. **c** Representative images of colonies formed by SUM159 shNC and shM14 cells treated with one-shot irradiation. **d** The m^6^A level of global mRNA in MDA-MB-231 cells overexpressing WT or mutated *METTL14* was determined by dot blotting. MB staining was used as a loading control. Data are presented as the mean ± SD for a representative of three independent experiments performed in triplicate. *P < 0.05; **P < 0.01.

**
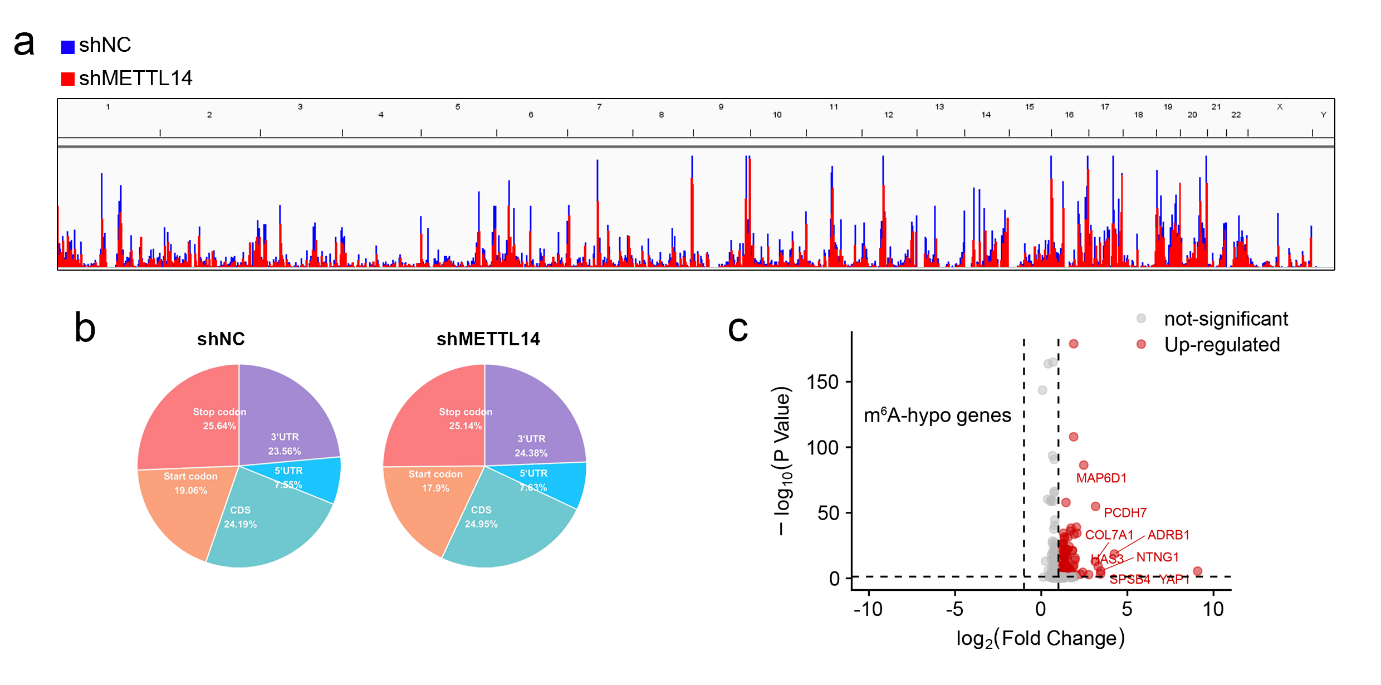
**

**Suppl. Fig. 3: Identification of m^6^A-regulated transcripts involved in TNBC stemness. a** IGV shows the m^6^A peaks identified in MCF7 shNC and shM14 cell lines. **b** Pie plots show the percentage of m^6^A distribution in the 5′ untranslated region (5’UTR), start codon, coding site (CDS), 3’UTR, and stop codon of mRNA in MCF7 shNC and shM14 cells. **c** Volcano plot shows the mRNA expression status of m^6^A-hypo genes in MCF7 cells upon *METTL14* depletion.

**
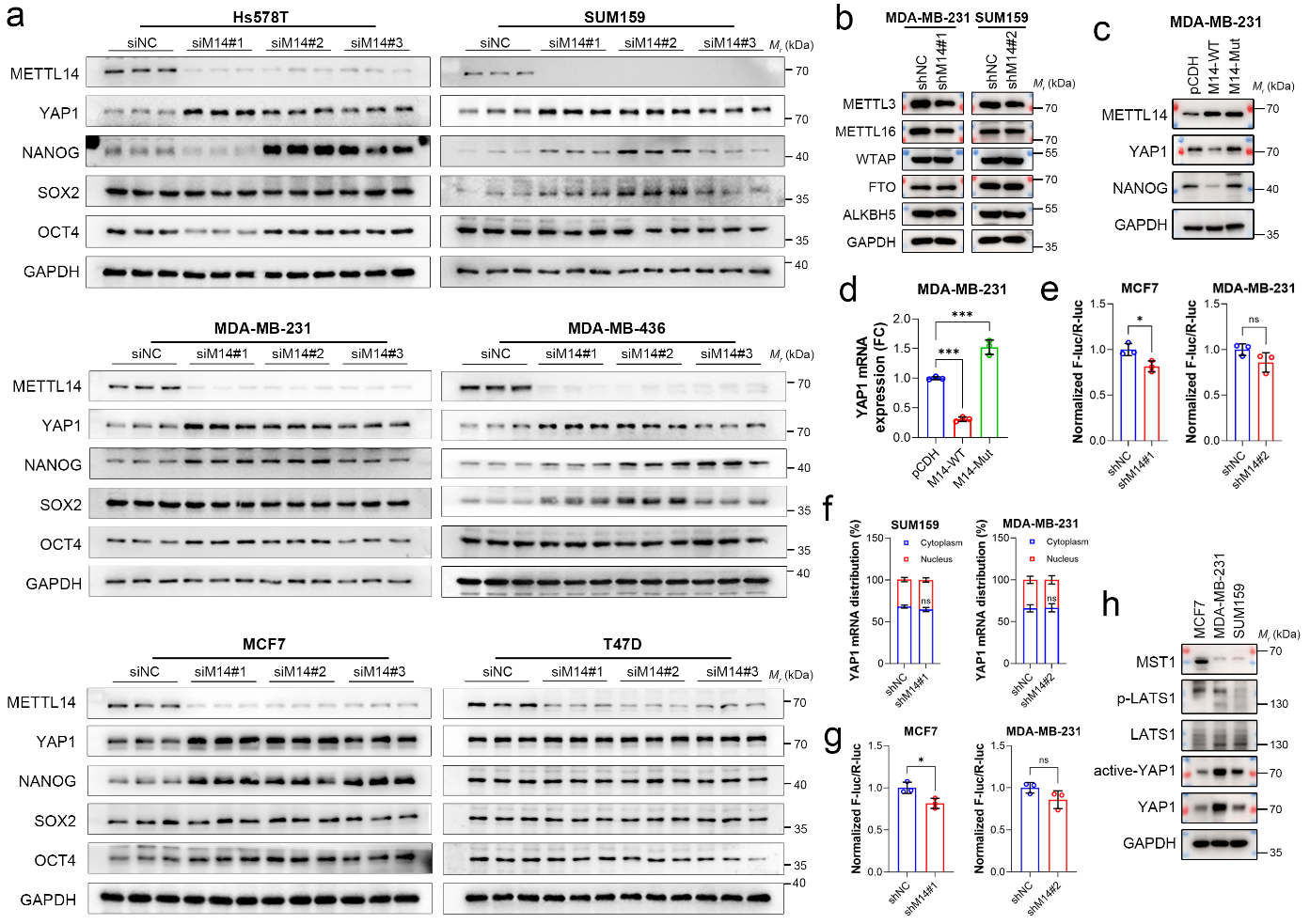
**

**Suppl. Fig. 4: METTL14 regulates *YAP1* expression independently of the Hippo signaling.** **a** Protein expression of METTL14, YAP1, NANOG, SOX2, and OCT4 in six BC cell lines treated with or without various siRNAs targeting *METTL14* was determined by immunoblotting. GAPDH was used as the loading control. **b** Protein expression of METTL3, METTL16, WTAP, FTO, and ALKBH5 in MDA-MB-231 or SUM159 cells with or without *METTL14* depletion was determined by immunoblotting. GAPDH was used as the loading control. **c** Protein expression of METTL14, YAP1, and NANOG in MDA-MB-231 cells overexpressing WT or mutated *METTL14* was determined by immunoblotting. GAPDH was used as the loading control. **d** The mRNA expression of YAP1 in MDA-MB-231 cells overexpressing WT or mutated *METTL14* was detected by qPCR. **e** The promoter region (-800/+100) of *YAP1* was cloned into the *pGL3-Basic* vector, which was then transfected into BC cell lines with or without *METTL14* depletion for 24 h. *YAP1* transcription activity was determined by dual-luciferase reporter assay. **f** Distribution of *YAP1* mRNA in the nucleus and cytoplasm was determined by qPCR. **g** The exon 10 and 3’UTR of *YAP1* were cloned into the *pmirGLO* vector, which was then transfected into BC cell lines with or without *METTL14* depletion for 24 h. The translation efficiency of F-luc was determined using dual-luciferase reporter assay and qPCR analysis. **h** Protein expression of MST1, p-LATS1, LATS1, active YAP1, and YAP1 in MCF7, MDA-MB-231, and SUM159 cells was determined by immunoblotting. GAPDH was used as the loading control. Data are presented as the mean ± SD for a representative of three independent experiments performed in triplicate. *P < 0.05; ***P < 0.001; ns, nonsignificant.

**
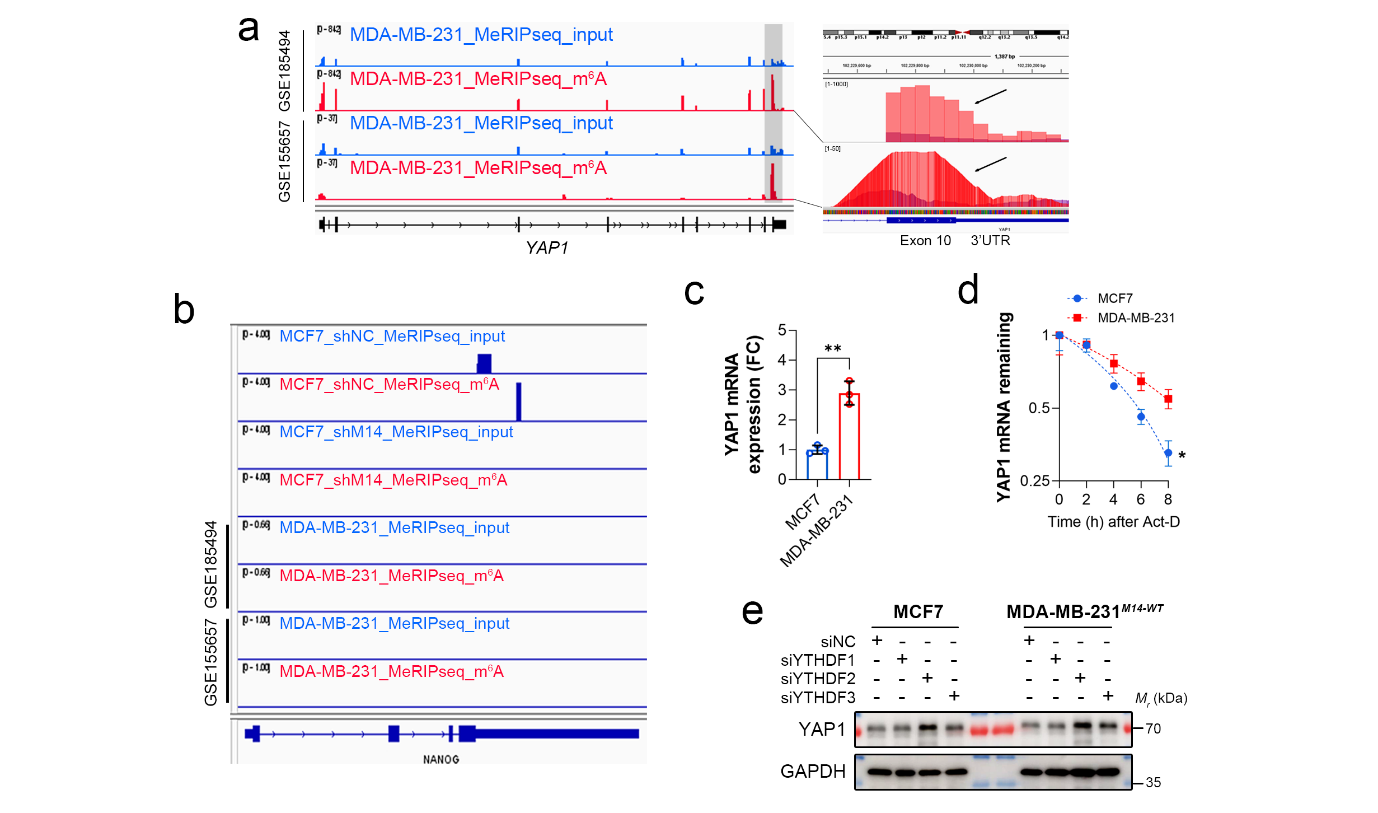
**

**Suppl. Fig. 5: The m^6^A-modified 3’UTR triggers YTHDF2-dependent YAP1 mRNA decay.** **a** IGV showing specific m^6^A signals and peaks in *YAP1* of MDA-MB-231 cells from the GSE155657 and GSE185494 datasets. The arrow indicates the m^6^A peak regulated by METTL14. The Y-axis shows the normalized read coverage. **b** The upper IGV shows m^6^A signals in the *NANOG* gene of MCF7 cells with or without *METTL14* depletion. The lower IGV shows m^6^A signals on the *NANOG* gene of MDA-MB-231 cells from the GSE155657 and GSE185494 datasets. **c** The mRNA expression of *YAP1* in MCF7 and MDA-MB-231 cells was detected by qPCR. **d** MCF7 or MDA-MB-231 cells were treated with 5 μg/mL Act.D for the indicated time. The stability of *YAP1* mRNA was determined by qPCR. **e** MCF7 and *METTL14*-overexpressing MDA-MB-231 cells were transfected with siNC, siYTHDF1, siYTHDF2, or siYTHDF3 for 48 h. The protein expression of YAP1 was detected by immunoblotting. GAPDH was used as the loading control. Data are presented as the mean ± SD for a representative of three independent experiments performed in triplicate. *P < 0.05; **P < 0.01.

**
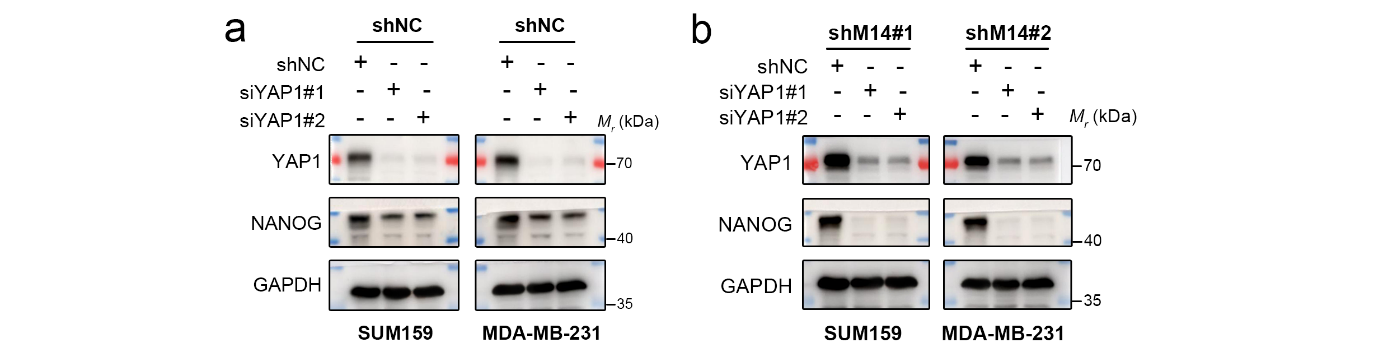
**

**Suppl. Fig. 6: YAP1 underlies the m^6^A-regulated stemness in TNBC.** **a** Protein expression of YAP1 and NANOG in SUM159 and MDA-MB-231 shNC cells with or without *YAP1* knockdown was determined by immunoblotting. GAPDH was used as the loading control. **b** Protein expression of YAP1 and NANOG in SUM159 and MDA-MB-231 shM14 cells with or without *YAP1* knockdown was determined by immunoblotting. GAPDH was used as the loading control.

**
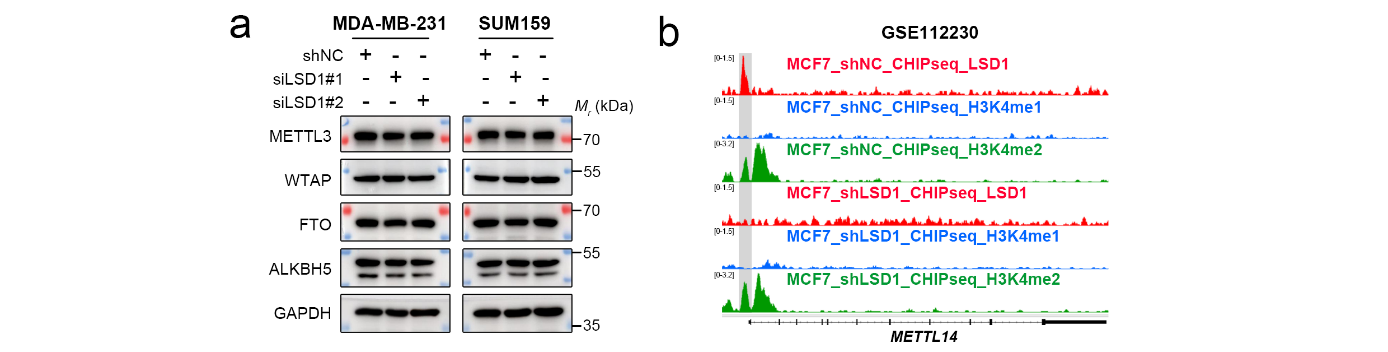
**

**Suppl. Fig. 7: The expression of *METTL14* is transcriptionally suppressed by LSD1.** **a** Protein expression of METTL3, WTAP, FTO, and ALKBH5 in MDA-MB-231 and SUM159 cells with or without *LSD1* knockdown was determined by immunoblotting. GAPDH was used as the loading control. **b** IGV visualization shows LSD1 and H3K4me1/2 signals at the *METTL14* promoter of MCF7 cells with or without *LSD1* knockdown from the GSE112230 dataset. The gray square indicates changes in LSD1 and H3K4me1/2 signals at the *METTL14* promoter due to *LSD1* knockdown. The Y-axis shows the normalized read coverage.
